# Antibody-Dependent Enhancement with a Focus on SARS-CoV-2 and Anti-Glycan Antibodies

**DOI:** 10.3390/v15071584

**Published:** 2023-07-20

**Authors:** Marina M. Ziganshina, Nadezhda V. Shilova, Eugenia O. Khalturina, Natalya V. Dolgushina, Sergey V. Borisevich, Ekaterina L. Yarotskaya, Nicolai V. Bovin, Gennady T. Sukhikh

**Affiliations:** 1 National Medical Research Center for Obstetrics, Gynecology and Perinatology of the Ministry of Health of the Russian Federation, Oparina Street 4, 117997 Moscow, Russia; pumatnv@gmail.com (N.V.S.); jane_k@inbox.ru (E.O.K.); ndolgush@gmail.com (N.V.D.); inter_otdel@mail.ru (E.L.Y.); secretariat@oparina4.ru (G.T.S.); 2 Shemyakin–Ovchinnikov Institute of Bioorganic Chemistry, Russian Academy of Sciences, 117997 Moscow, Russia; professorbovin@yandex.ru; 3 Federal State Autonomous Educational Institution of Higher Education I.M. Sechenov First Moscow State Medical University of the Ministry of Health of the Russian Federation (Sechenov University), 119991 Moscow, Russia; 4 Russian Academy of Sciences, 109240 Moscow, Russia; sp_borisevich@mail.ru

**Keywords:** antibody-dependent enhancement, natural antibodies, anti-glycan antibodies, COVID-19, SARS-CoV-2, virus-neutralizing activity

## Abstract

Antibody-dependent enhancement (ADE) is a phenomenon where virus-specific antibodies paradoxically cause enhanced viral replication and/or excessive immune responses, leading to infection exacerbation, tissue damage, and multiple organ failure. ADE has been observed in many viral infections and is supposed to complicate the course of COVID-19. However, the evidence is insufficient. Since no specific laboratory markers have been described, the prediction and confirmation of ADE are very challenging. The only possible predictor is the presence of already existing (after previous infection) antibodies that can bind to viral epitopes and promote the disease enhancement. At the same time, the virus-specific antibodies are also a part of immune response against a pathogen. These opposite effects of antibodies make ADE research controversial. The assignment of immunoglobulins to ADE-associated or virus neutralizing is based on their affinity, avidity, and content in blood. However, these criteria are not clearly defined. Another debatable issue (rather terminological, but no less important) is that in most publications about ADE, all immunoglobulins produced by the immune system against pathogens are qualified as pre-existing antibodies, thus ignoring the conventional use of this term for natural antibodies produced without any stimulation by pathogens. Anti-glycan antibodies (AGA) make up a significant part of the natural immunoglobulins pool, and there is some evidence of their antiviral effect, particularly in COVID-19. AGA have been shown to be involved in ADE in bacterial infections, but their role in the development of ADE in viral infections has not been studied. This review focuses on pros and cons for AGA as an ADE trigger. We also present the results of our pilot studies, suggesting that AGAs, which bind to complex epitopes (glycan plus something else in tight proximity), may be involved in the development of the ADE phenomenon.

## 1. Introduction

Coronavirus disease 2019 (COVID-19), caused by severe acute respiratory syndrome coronavirus 2 (SARS-CoV-2), resulted in more than 767 million illnesses worldwide and more than 6.9 million deaths as of 17 June 2023 according to WHO (WHO. WHO Coronavirus (COVID-19) Dashboard (2022). Available online: https://covid19.who.int/ (accessed on 17 June 2023). Some data show that severe COVID-19 disease may be complicated by antibody-dependent enhancement (ADE) [1,2]; this phenomenon was found in patients with SARS-CoV and MERS-CoV, respiratory syncytial virus (RSV), dengue [3,4], influenza [5], HIV, and viral diseases in animals [2,6]. It is difficult to specify whether severe COVID-19 is caused by ADE or other factors. The reason is the lack of diagnostic criteria for ADE, since there are no data on the involvement of specific antibodies in the pathogenesis of ADE-associated infection enhancement.

SARS-CoV-2 is a single-stranded RNA virus with four structural proteins: small envelope (E), matrix (M), nucleocapsid (N), and spike (S). Antibodies to SARS-CoV-2 proteins and unique antigenic peptides were found in patients with COVID-19 [7,8,9,10,11]. The transmembrane S-protein is extensively glycosylated with a total of 22 potential sites of N-glycosylation and several O-linked glycosylation sites which mediate infectivity and immune escape [12,13,14]. Pre-existing antibodies to glycans recognize the virus and potentially influence disease progression [15]. In some infections [16,17,18], natural anti-glycan antibodies (AGA) were found to contribute to the disease development. A previously studied AGA profile in patients with COVID-19 indicates that the abnormally high IgG and IgM antibodies observed against number of self-glycans may help explain some of the unusual and prolonged symptoms observed in severe COVID-19 patients [15]. The natural AGAs are also associated with activation of the antiviral immune response [19]. However, there is no clear evidence of their role in COVID-19. Considering that AGAs apparently account for up to half of natural antibodies [20], it would be important to find out if they play a role in the activation of the antiviral immune response in COVID-19.

## 2. Terminological Debate

In many ADE studies, the antibodies which had emerged as an immune response in previous viral infection are called *pre-existing*; on the other hand, the same term is commonly applied elsewhere to natural antibodies, i.e., those that are initially present in the human body (and are not a straight reaction to non-self antigens). Therefore, to avoid confusion, we suggest to name infection-induced cross-reactive antibodies as *already existing antibodies* (AEAbs).

As summarized in several reviews (e.g., [1,6,21,22]), the ADE researchers consider that ADE-associated antibodies are AEAbs, i.e., the antibodies which had emerged in response to previous infection and are still present by the time of a new infection. However, a homologous or heterologous viral genotype causes a new generation of virus-specific antibodies, the role of which in ADE is difficult to determine for several reasons. Firstly, the time of the secondary immune response to the homologous antigen (including the B-cell response) is significantly shorter than the primary response. This creates great methodological difficulties in distinguishing AEAbs from secondary antibodies due to the lack of adequate laboratory tests. Moreover, the second generation of antibodies has higher affinity than AEAbs, which titer decreases by the time of reinfection, and the pool comes represented by antibodies with lower affinity; therefore, the participation of a “new portion” of antibodies in ADE is less probable than the involvement of AEAbs. Finally, the time of generation of antibodies to heterologous epitopes is about 21 days; for this reason, the antibodies with mature affinity do not reach a neutralizing concentration by the time of the infection enhancement. These arguments do not completely exclude a probability of contribution of the newly synthesized antibodies to the ADE phenomenon but support the AEAbs as ADE-associated, and in our review, they will be considered in this context.

The next debatable point is the use of the term *subneutralizing antibodies* by ADE researchers. This term seems improper for antibodies of primary or secondary immune response to homologous or heterologous viral epitopes but can be used for cross-reactive AEAbs with low affinity/low avidity which are present in the blood at “below neutralizing” concentrations. These antibodies provide only partial binding of viral epitopes, resulting in incomplete blocking of receptor-binding domains of viral protein [23,24], and their strength of interaction with the epitope is below a certain threshold [25,26]. To avoid ambiguous interpretations, the antibodies with such characteristics should be defined as “antibodies with weak virus neutralizing activity”.

A significant part of this manuscript is devoted to AGAs and their role in the antiviral immune response in COVID-19. Since there is no reliable way to differentiate between natural and adaptive AGAs, from now on we will consider all AGAs as a part of the overall immunoglobulin pool of a healthy donor.

## 3. Antibody-Dependent Enhancement (ADE): Phenomenon Outline

ADE is a phenomenon where virus-specific antibodies resulting from a prior infection, vaccination, or passive transfer, including treatment with hyperimmune plasma of convalescents, are able to prevent infection and, moreover, contribute to its spread with severe complications. Antibodies that bind to the virus but have no neutralizing effect (non-neutralizing antibodies) or have a weak neutralizing effect (antibodies with weak virus neutralizing activity) due to their insufficient concentrations and/or low affinity/avidity are considered to cause the ADE phenomenon in viral infections [27].

There are three main mechanisms of the ADE phenomenon with some variations:

Mechanism 1: AEAbs bound to the virus through the Fc fragment interact with the Fc receptors (FcRs) of immune cells (macrophages and other phagocytic immune and non-immune cells—in particular, epithelial cells) and promote the pathogen penetration into cells by means of endocytosis [28,29]. The development of ADE through this mechanism is thought to result in the increased replication of the virus and its intense spread in the body. The clinical phenotype of ADE developing through this mechanism is more severe and is characterized by a prolonged disease [1,21,30]. 

Mechanism 2: AEAbs bind to the virus and activate the complement system. Through interaction with complement receptors (CRs), the resulting complex penetrates phagocytic immune cells and other types of cells—in particular, smooth muscle, follicular dendritic, B cells, fibroblasts, and endothelial cells [31]. A version of this mechanism is penetration of the virus–antibody complex into the target cell through the components of the complement system: C1q or C3 [1,2,32,33]. This process activates excessive immune response, initiated by immune complexes inside airway tissues, resulting in the secretion of proinflammatory cytokines, immune cell recruitment, and activation of the complement cascade within lung tissue [2,4]. The antibody-dependent mechanism of pathogen penetration into the cell is the most simple and effective, and therefore, under certain conditions, prevails over the mechanism involving the virus-specific receptor. 

Mechanism 3: AEAbs-induced change in the conformation of the viral protein, which promotes the fusion of the viral envelope with the membrane of the target cell [28]. 

All described mechanisms, based on the antibodies-mediated penetration of the pathogen into the target cell, lead to the spread of the infection in the body and to hypertrophic immune responses. Clinically, ADE manifests with deterioration of the patient’s condition after the onset of the disease. In severe cases, it leads to tissue damage and multiple organ failure. The cause is uncontrolled innate inflammatory responses and impaired acquired immune responses resulting in severe inflammatory response, increased tissue infiltration by macrophages and neutrophils, and cytokine storm, establishing an aberrant inflammatory feedback loop with a decrease of lymphocytes, CD4+ T cells, CD8+ T cells, B cells, and NK cells [2,34,35,36,37].

## 4. ADE in Viral Infections: Paradoxes and Key Debatable Issues

### 4.1. Dengue Fever Is a Classic Example of ADE

The most severe complications of ADE were seen in dengue fever, which is the most demonstrative example of this phenomenon. Dengue virus (RNA-genomic group B arbovirus, genus Flavivirus, family Togaviridae), causes classic dengue fever and clinically severe disease with complications (viral hemorrhagic fevers, dengue shock syndrome) [38,39]. To date, four dengue virus genotypes sharing approximately 65% homology are known: DENV1, DENV2, DENV3, and DENV4 [40,41]. The viruses of these genotypes stimulate the production of genotype-specific antibodies with high neutralizing activity and short-term cross-neutralizing activity against heterologous viral genotypes [41,42]. Reinfection with a different viral genotype often causes a more severe course of the disease due to the presence of antibodies produced during the primary infection. These antibodies contribute to the entry of the pathogen into cells and make the symptoms more severe [21,30]. A paradox occurred with the first live-attenuated chimeric yellow-fever/tetravalent dengue vaccine (CYD-TDV), Dengvaxia, which showed low effectiveness and, conversely, increased the risk of severe dengue fever when infected with DENV of other genotypes [43].

The paradox of immune responses in vaccinated people or in those who have been re-infected with a different virus genotype is that, in addition to the receptor-mediated endocytic pathway for viral entry into the target cells, another mechanism develops: entry of the antibody-opsonized DENV in the ADE condition follows a phagocytosis pathway into the macrophages, monocytes, or the DCs by means of FcγR crosslinking [44,45,46]. However, not only the Fc receptors contribute to DENV entry into cells. Other cell surface molecules such as glycosaminoglycans (GAG), lipopolysaccharide-binding CD14 associated molecules, heparan sulfate, and lectin-like receptors, such as DC-SIGN (dendritic cell-specific intercellular adhesion molecule 3-grabbing non-integrin), are also involved in the receptor-mediated endocytosis [42]. The envelope (E) protein, (pre) membrane protein (prM/M), and nonstructural (NS) proteins of the virus, particularly NS1, NS2A, NS2B, NS3, NS4A, NS4B, and NS5 DENV, were found to be glycosylated, and glycosylation differs between genotypes [47,48]. N-glycosylation on both E and NS1 proteins has been shown to play important roles throughout the DENV infection cycle from virion attachment, entry, maturation, and assembly to secretion [49]. Carbohydrate–protein interactions between glycans and endogenous lectins of DENV and host have already been proven to affect the virulence and immunogenicity of the virus, but the role of this type of interaction for the development of ADE has not been specified [49,50]. However, given that the inhibitory effects of lectins on virus attachment and penetration into mammalian and mosquito cells have been shown experimentally, it is possible to assume the involvement of AGA in these processes since they, like lectins, are carbohydrate-binding proteins.

After penetration into the cell, the virus activates a number of genes that promote replication, DENV mRNA processing, and vesicle transfer, simultaneously suppressing the expression of early anti-inflammatory response genes [51,52]. DENV negatively regulates Th1 response and IFN-γ production but stimulates IL-6 and IL-10 synthesis; this causes an anti-inflammatory effect with the dominance of Th2 responses [42,53], contributing to B-cell proliferation and antibody hyperproduction, which enhance ADE development [45,46]. The clinical manifestation of this phenomenon is raising a number of questions, which are difficult to answer.

### 4.2. Can Virus-Specific Antibodies Intensify Infection Instead of Protecting against It?

This is the main puzzling question which could not be answered at the beginning of the studies of this phenomenon, since the latter did not fit the concepts of classical immunology. The activation of the infection by antibodies is paradoxical, since it is believed that the AEAbs should act as a protective factor in recurrent disease.

### 4.3. What Is the Role of ADE in the Infection Enhancement?

Another paradox of ADE is that this phenomenon may be suggested only with exacerbation of the clinical signs of infection, since there are no direct molecular markers of ADE or predictors of the severe course of the disease. Therefore, a number of reviews doubt that ADE plays a real role in the infection enhancement [54,55]. In a number of infectious diseases, particularly those caused by dengue virus, ADE can be diagnosed in seropositive patients with antibodies detectable by commercial tests [56,57]. ADE development depends on the titers of already existing antibodies capable of interacting with viruses [58]. In a number of other infections, including those caused by coronaviruses, ADE can hardly be captured in vivo but can be confirmed in cell cultures [25,59,60]; these tools are quite useful for in vitro studies [21,27]. For example, a cell system, which was introduced as a model framework for ADE, allows to study the molecular mechanisms of coronavirus penetration (MERS-CoV, SARS-CoV) into cells expressing Fc receptors using RBD-specific neutralizing MAbs [28]. However, these data cannot be applied to clinical cases of infection, since it is not possible to prove the role of antibodies of a certain specificity in the development of ADE in vivo [61,62]. Nevertheless, in COVID-19, the presence of antibodies to other coronaviruses and the low level of AEAbs to SARS-CoV-2 after primary infection are considered as a risk factor for ADE in subsequent infections [63].

### 4.4. What Factors Trigger the Development of ADE?

To date, there is no consensus on whether there is one key factor that triggers ADE or a combination of several factors. The most often mentioned factors for the development of ADE are

- the type of the infected cell’s Fcγ receptor to which the antibodies bind;
- G class antibody isotype;
- specific glycosylation, which differs from the glycosylation of the antibodies of the same epitope specificity which do not cause ADE [59,63,64,65,66,67,68,69,70,71];
- concentration of antibodies [23,24];
- epitope specificity of antibodies.

Of all these factors, only the latter is realized through the Fab fragment and determines two functional aspects of antibodies:

(i) interaction of the immunoglobulin molecule with specific epitopes of a particular virus should normally lead to virus neutralization and block its penetration into the cell, but in ADE, this process is characterized by incomplete neutralization and activation of effector cells;
(ii) antibodies binding to homologous epitopes of heterologous virus genotypes do not neutralize the virus but only provide a binding effect and may be involved in antigen masking or effector reactions [72].

Other factors are related to the interactions that are mediated through the Fc fragment, to the ability of the virus to induce the production of antibodies of a certain subclass, and to alter the metabolic and biosynthetic pathways in the cell [73,74,75,76,77]. However, all of the mentioned factors must be considered together because the affinity and binding specificity of the Fc domain for different FcγRs are determined by differences in the primary amino acid sequence of the IgG subclasses (IgG1–IgG4 in humans), as well as by the structure and composition of the Fc-associated glycan structure (or, more broadly, post-translational modification). These two determinants drive Fc domain diversification, resulting in IgG Fc domains with different capacities for engaging and activating the various members of the FcγR family expressed by effector leukocytes [65]. Nevertheless, it seems that antibody specificity is the main and determining factor of ADE development since the effector responses mediated by Fc fragment are secondary, and interactions are activated through a specific pathway. Thus, it is necessary to identify the specificity of ADE-associated antibodies and study their antigen-binding properties.

### 4.5. Which Antibodies Are Associated with ADE?

The subtle epitope specificity of antibodies causing ADE remains undetermined for all viral infection. The only sign of ADE is the presence of AEAbs, which are targeted to viral surface protein fragments. However, these antibodies are also considered to be protective (neutralizing antibody), since their presence is an evidence of the immunity strength after illness or vaccination. Therefore, the criteria for distinguishing antibodies as potentially causing ADE should be defined. 

The definition of ADE phenomenon (given in the beginning of this manuscript) indicates that antibodies involved in this phenomenon cause the effect opposite to virus neutralization. Both ADE and virus neutralization require the binding of the antibody to the virus. But in the case of ADE, binding to the viral epitope is either (i) incomplete (not all epitopes are blocked) or (ii) not effective for virus neutralization. These two points are most important and must be taken into account when formulating criteria determining ADE-associated antibodies. 

The neutralizing activity of antibodies is determined by the affinity of their interaction with epitopes of viral proteins and the strength of cooperative interactions [78]. This means that through high-affinity/avidity interactions of antibodies with the virus, the latter is neutralized by blocking its binding to specific cell receptors or by fusing the viral membrane and membrane of the target cell [79]. The resulting complex is absorbed by phagocytes; in their cytoplasm, proteolytic cleavage and deproteinization of the virus take place, followed by the activation of antigen-presenting cells and adaptive immunity cells and the development of a specific immune response with stimulation of synthesis of a new generation of antibodies with higher affinity/avidity. This is the way the virus is neutralized and removed, along with the stimulation of the synthesis of protective virus neutralizing antibodies in the classical antiviral immune response [2,80].

Another paradox and serious challenge in ADE research is that antibodies to the same epitope cause different effects, depending on their concentration in blood [57,81]. This dependence, which determines the antiviral activity of antibodies or causes incomplete neutralization and/or intensification of infection, has been shown in in vitro studies where primary cultures of myeloid cells and continuous cell lines expressing Fc receptor or virus-specific receptor were co-cultured. The addition of polyclonal or monoclonal antibodies to peptide fragments of SARS-CoV-2 RBD in various dilutions has confirmed an amount-dependent effect (high concentrations of antibodies prevent cell infection). However, in a certain “window” of concentrations, the neutralizing antibodies can intensify infection, and therefore, they are considered to be infection-enhancing antibodies [21,28]. 

The data from various model systems of ADE studies suggest that in low affinity/avidity interactions of antibodies with viral epitopes, the virus–antibody complex decomposes after entering the cell and initiates virus replication, inflammation, and tissue damage by activating myeloid cells through their FcRs [59,82]. For physiological effect (neutralization), the antibody concentration must be low when the affinity/avidity to the receptor is high, and conversely, when the affinity/avidity is low, the antibody concentration must be high to maximize capture of receptors [25,63,78,82,83,84]. However, when the concentration of antibodies (especially low affinity/avidity antibodies) decreases, the virus neutralization becomes ineffective; therefore, antibodies convert from neutralizing to weakly neutralizing, thus meeting the criterion “(i)” described above. In this case, even a small amount of bound antibodies expresses the Fc fragment that can bind to the FcRs of phagocytic cells. Antibodies are produced in parallel against different epitopes. Each of them alone is not enough to neutralize the virus, but different antibodies act collectively to bind through the Fc receptor, which results in the penetration of the virus into the cell and the development of ADE [https://www.deplatformdisease.com/blog/what-is-antibody-dependent-enhancement-ade] (accessed on 1 June 2023) [28,85,86,87].

The studies of the immune response to dengue and Zika viruses have shown that a primary infection leads to the production of a wide range of antibodies, which contributes to the severity of the secondary infection. If the concentration of high avidity antibodies is high by the time of the secondary infection, a neutralizing antibody response develops to protect against the viral invasion. Low titers of neutralizing antibodies cause either negligible effects in mild or asymptomatic secondary infections with low or medium concentrations of medium/low avidity antibodies or lead to severe ADE with poor concentrations of low avidity antibodies [88]. 

Weakly neutralizing antibodies are believed to target the essential viral epitopes located in the receptor-binding domains (RBD) of the surface proteins which mediate virus reception and entry [28,87]. However, the antiviral response also leads to the production of antibodies to other epitopes, including those located outside the RBD [81,85,87,89]. These are often fragments of homologous areas that are identical in different viruses. Antibodies to these epitopes may be present in low titers in blood long after the disease, constituting a pool of AEAbs, some of which have low affinity/avidity for new genotypes. These immunoglobulins can bind to the virus, attract immune cells, move to the cytoplasm, and then release from the complex, initiating viral replication [90], thus meeting the “ineffective” binding criteria. Such antibodies are considered non-neutralizing, i.e., they bind the epitope but do not prevent viral replication [1]. The binding of these antibodies to viral epitopes meets the criterion “(ii)” above.

In addition to weak and non-neutralizing antibodies involved in the pathogenesis of ADE, a number of studies mention cross-reactive antibodies [89], i.e., antibodies that can interact with a number of different epitopes. In the context of ADE, this term is applied to antibodies belonging to AEAbs, which were produced due to vaccination or infection with a highly homologous virus of another serotype/other genus but of the same family [21]. Cross-reactive antibodies may show no virus neutralizing activity when they bind to RBD fragments that are not critical for the virus or to outside epitopes. Therefore, the function of these cross-reactive antibodies in some part confusingly overlaps with the function of the weak or non-neutralizing antibodies. In particular, a review by Pang NY et al. (2021) described that a number of neutralizing SARS-CoV-2 monoclonal antibodies taken from convalescents are cross-reactive to other epitopes outside the RBD area [91,92]. A big issue is an ambivalent performance of cross-reactive antibodies: their virus-neutralizing activity (VNA) is realized at high concentrations and mostly in a mix with other antibodies with stronger virus-neutralizing activity. But at low concentrations, and due to their cross-reactivity (which may be a result of an infection by a closely related virus), these antibodies can be classified as AEAbs and promote infection-enhancing reactions [63]. Therefore, the criteria for ADE-associated antibodies are rather vague and non-specific. All of the above demonstrates that there is no understanding on how the development of ADE can be predicted since the type of antibodies to be monitored in patients for this purpose has not been determined.

## 5. Infection Caused by SARS-CoV-2: Does ADE Develop?

Only three types of coronaviruses can cause extensive lung damage, namely betacoronaviruses SARS-CoV, MERS-CoV, and SARS-CoV-2 [93,94]. The development of ADE after SARS-CoV and MERS-CoV (structurally homologous to SARS-CoV-2) and after vaccination was confirmed in vitro, in vivo and in humans [95,96,97]. It was found that in SARS-CoV and MERS-CoV infection, ADE can develop when the virus enters the cell due to conformational changes of the spike protein as a result of binding to antibodies and subsequent interaction of immunoglobulin Fc fragments with FcRs [1,98,99]. There is limited evidence that ADE develops after SARS-CoV-2 infection and contributes to the severe course of COVID-19.

SARS-CoV-2 (in particular, the RBD of the S-protein which is essential for receptor binding) is subject to mutations [100,101]. As a result, many strains and genotypes of SARS-CoV-2 which cause reinfection in the presence of AEAbs have already been described [102]. ADE is hypothesized to be one of the causes of the severe course of COVID-19, especially of acute respiratory distress syndrome, the main cause of lethal outcome in COVID-19 [25,60,103,104,105]. To date, the low level of AEAbs after coronavirus infection, including other COVID-19 genotypes, is considered a main risk factor and probable trigger for ADE development in COVID-19 [2,63]. Experiments on cell lines showed that when bound to homologous epitopes of the new SARS-CoV-2 genotype, AEAbs amplified infection [106] by infecting CD32+-positive cells and facilitated virus proliferation in lung epithelial cells and tissue-infiltrating macrophages [25,60,105,106]. Antibodies (IgG) to low pathogenic human coronaviruses NL63 and OC43 were found to have cross-reactivity with SARS-CoV-2 in persons who have not previously been infected with this pathogen [107]. SARS-CoV and, to a less extent, MERS-CoV viruses lead to the production of cross-reactive antibodies to SARS-CoV-2 [66,108,109], but there are no evidence-based clinical studies showing the impact of these antibodies on infection amplification in COVID-19.

Another possible trigger of ADE is the transfer or production of antibodies as a result of passive immunization or vaccination [85]. However, the passive immunization of patients with severe course of the disease with hyperimmune plasma from COVID-19 convalescents did not induce ADE but instead was effective and safe for treatment of COVID-19 [85,110]. Despite a significant number of studies that doubt the development of ADE in severe COVID-19, others demonstrate high probability of this event.

## 6. Anti-Glycan Antibodies as a Possible ADE Trigger in COVID-19: Pros and Cons

### 6.1. Do SARS-CoV-2 Glycans Induce an Immune Response?

An important factor that may influence the development of the ADE phenomenon is the *Heavy* glycosylation of viral proteins. Glycosylation mapping of the spike protein subunits revealed a variety of O-linked and N-linked glycans, including high-mannose [111]. Due to the peculiarities of the virus reproduction (they use the host cell for glycosylation of their own proteins), the composition of viral glycans does not significantly differ from the glycans of the host cell [49,112,113]. The glycans of the RBD of SARS-CoV-2 spike glycoprotein were characterized in detail. However, it should be noted that all structural studies of glycans were carried out for viruses grown in cell cultures—that is, there is no guarantee that real viruses of an infected patient (for obvious reasons, inaccessible for structural studies) have exactly the same glycans as viruses grown in a culture of human (and even moreover, in the other) cells. Terminal N-acetyllactosamine (LacNAc) units were identified and appeared to be decorated with α2,3- or α2,6-linked sialyl moieties. In addition to N-bound, the glycoprotein also contains O-bound sialylated and nonsialylated glycans: HexNAc_1_Hex_1_NeuAc_2_ > HexNAc1Hex_1_ > HexNAc_1_Hex_1_NeuAc_1_ > HexNAc_2_ > HexNAc_1_ > HexNAc_2_Hex_1_ (Hex is hexose) [114]. Terminal GalNAcβ1-4GlcNAc (LDN) was found, along with their α2,6-sialylated and 4-O-sulfated derivatives (6′SLDN and 4SulLDN) [115]. These structural motifs, along with terminal LacNAc, LDN, 3′SLN and 6′SLN fragments, are predominant epitopes on the outer chains of the RBD N-glycan [115]. Such glycans are widely represented (except for LDN, see below) in human cells [116]. The identity of the viral glycans and host glycans explains the ability of viruses to evade from cell and humoral effectors of innate and adaptive immunity [112,113,117]. One of the mechanisms of evasion of viruses from the immune system is the interaction between the sialoglycans, which are found in the RBD of SARS-CoV-2 spike glycoprotein, and siglecs, endogenous lectins expressed by cells of innate and adaptive immunity; this interaction leads to a late immune response to the virus [118]. The study cited above [115] showed the presence of a high degree of fucosylation, both at the core and at terminal positions, corresponding to Lewis X (LeX) and fucosylated LDN (LDNF). The finding of LDN and LDNF was somehow unexpected as it has usually been related to parasites, and was thought to cause immunogenic response in humans [119,120,121]. In the vast majority of cases, viral glycans are recognized as intrinsic, which prevents the development of an immune response to these glycans [122,123]. As a result, AGA do not contribute to the antiviral immune response induced by specific antibodies that bind to the pathogen protein. However, the example given above indicates that antibodies can still be generated against a number of glycans and, possibly, not only against “pure” glycotopes but also against glycopeptides. A study of neutralizing monoclonal antibodies showed that the S309 antibodies can bind to a protein/glycan epitope on SARS-CoV-2 RBD [66,92], which proves the involvement of antibodies that specifically bind glycans in the antiviral immune response.

### 6.2. Anti-Glycan Antibodies as a Pool of Antibodies with Antiviral Activity

The pool of antibodies in a healthy person is represented by natural antibodies (these appear without any collision with antigens and can be detected in the first months of life) and adaptive antibodies (an outcome of an immune response to an antigen and reflection of immunological history). ADE studies usually focus on adaptive antibodies, while natural antibodies are often ignored, even though they are the true pre-existing antibodies.

Natural antibodies make up a significant part of the antibodies pool, and many of them are directed to glycans. Humans have a diversity of AGA specificities and a significant number of AGA of some specificities [20,124,125,126], which remains constant throughout life [20]. Changes of AGA concentrations, increased or decreased, are indicative of the development of pathology [20,125,126].

The natural antibodies perform well known regulation and control functions [124,127,128,129,130,131]. They are known to bind to a significant number of PAMPs (pathogen-associated molecular patterns), which are structural units of bacterial and viral antigens, and indicate the extrinsic and infectious nature of the agent. Their binding to PAMPs is realized through the neutralization and elimination of the latter. This process is a result of the effector reactions of the immune system, which include phagocytosis with preceding complement activation, induction of antibody-dependent cellular cytotoxicity, involvement in antigen presentation to T cells, and stimulation of the humoral immune response [132,133]. Natural antibodies and AGA, as a part of their pool, have a potential to activate the mechanisms of ADE development, including those involving complement.

We formulated the following assumptions that support the involvement of AGA in the pathogenesis of the ADE phenomenon:

(i) AGA are part of the pool of natural pre-existing antibodies and are able to recognize virus-associated glycans;
(ii) Most of AGA are polyreactive antibodies, i.e., they can bind to a significant number of epitopes due to the conformational lability of epitopes and paratopes of the antibodies [134]. The broad epitope specificity of AGA determines their binding not only to carbohydrate antigens but also to glycolipid and glycopeptide antigens [135];
(iii) A significant number of AGA have low affinity compared to protein–protein interactions. Their affinity for the monovalent hapten (in K_D_ terms) is in the range of 10^–4^–10^–6^ M. AGA content of some specificities (this refers to “top” antibodies) in healthy persons reaches the level of ~0.5% of the total amount of immunoglobulin M class [20], which indicates their rather high concentration in blood.

## 7. Anti-Glycan Antibodies as ADE Trigger

### 7.1. Involvement of Anti-Glycan Antibodies in ADE Development in Bacterial Infections

It has been found that AGA binding to the polysaccharides of *S. pneumoniae*, *S. aureus*, and the toxin from *B. anthracis* can intensify the infection and compete with the protective antibodies produced by immunization (particularly against *S. aureus*), leading to the development of disease complications [16,17]. A paradoxical exacerbation of the infection caused by *N. meningitidis* and bacteria of the *Enterobacteriaceae* family was also detected for antibodies to the so-called Galα-epitope, Galα1-3Galβ1-4GlcNAc. The antibodies are found in significant amounts in the blood of all humans and are the main antibodies responsible for organ rejection in xenotransplantation [18,136,137]. αGal antibodies have been shown to bind to certain pathogenic intestinal bacteria, which cause sepsis more strongly, than to normal microflora. Additionally, under certain conditions, these antibodies participate in reactions that support inflammation. Their binding to pathogenic bacteria is associated with the resistance of bacteria to the factors of natural and adaptive immunity. An in vivo experiment simulating sepsis in α1,3 galactosyltransferase knockout mice showed better survival rates of mice in which anti-αGal Abs have been removed. Positive outcomes were associated with a bactericidal effect due to the increased binding of peripheral blood IgG to *Escherichia coli* isolates and reduced anti-inflammatory cytokines in mice [138]. The increased efficiency of humoral immunity and decreased sepsis mortality after the removal of anti-αGal antibodies from blood indicates their relation to antibodies causing ADE. 

### 7.2. Anti-Glycan Antibodies in Coronavirus Infection: Data from Previous Studies

#### 7.2.1. Autoantibodies Induction in Coronavirus Infection

SARS-CoV and SARS-CoV-2 coronaviruses have been shown to cause the production of autoantibodies; in particular, in SARS-CoV-2, the antibodies to major viral antigens (S- and N-proteins) are cross-reactive to human proteins [139]. In animals, after immunization with a vaccine derived from inactivated and purified SARS-CoV virus, antibodies targeted to normal cell components, including the carbohydrate chains of human orosomucoid, were detected [117]. Antiglycan antibody testing performed with the 816-printed glycan array (PGA) revealed antibodies (IgM and IgG) to numerous self-carbohydrates, including gangliosides, N-linked glycans, LacNAc derivatives (particularly LNnO), blood group H (type 1) antigen, and sialyl Lewis X in COVID-19 patients, which are not found in healthy individuals [15]. It is assumed that the above-mentioned antibodies contribute to the autoimmune reactions in COVID-19 and are responsible for the long-term post-COVID-19 syndrome. In particular, antibodies to gangliosides are associated with neurological complications in COVID-19 patients. In addition, there was an increase in antibodies for a variety of other glycans, including Lewis C/Sialyl Lewis C, rhamnose, the Forssman antigen, and several others, although the increase was not dramatic in terms of both magnitude and incidence among patients [15]. These findings are supported by our own studies of AGA in patients with COVID-19 of varying severity compared to healthy donors using a similar approach (presented below). 

#### 7.2.2. AGA Virus-Neutralizing Activity

In a cohort study [140] the risks of SARS-CoV-2 transmission in married couples with various AB0 blood types were studied. The risk of viral transmission was found to be minimal when blood types were different and maximal in couples with compatible blood types. It was assumed that natural AGA to AB0 antigens are involved in viral neutralization. This was later confirmed in the studies [19,141,142,143,144], which demonstrated the VNA of anti-A antibodies against a number of enveloped viruses. It was shown that the interaction between S-protein (SARS-CoV-2) and ACE2 in cell cultures was dose-dependently blocked by anti-A antibodies, provided that the S-protein was synthesized by cells that expressed an A antigen. This finding is also supported by the low levels of anti-A and anti-B alloantibodies detected in patients with COVID-19 compared to healthy individuals [145], since the depletion of these antibodies is associated with their implemented virus-neutralizing activity. It is assumed that the resistance to SARS-CoV-2 depends on the titers of the anti-A and anti-B antibodies which vary considerably between the individuals in the population. Thus, the AGA of certain specificities may inhibit the viral spread [144]. 

The structural similarity between the A blood type antigen and the Tn (GalNAcα) antigen (whose critical motif is the GalNAcα residue attached to the polypeptide chain) stimulated a study of the relevant antibodies in patients with COVID-19 [19]. Tn antigen is a tumor-associated marker in a number of cancers, formed as a result of intensive biosynthesis in a transformed cell, when glycosylation lags behind the synthesis of the polypeptide chain. It has been shown that the S-protein of coronavirus contains O-chains including the Tn antigen [114]. Antibodies to this antigen are present in almost all people, regardless of blood type, but their level and fine epitope specificity change significantly with the neoplastic process, and could be used as a prognostic marker [146]. The levels of anti-Tn antibodies was shown to be significantly lower in patients with COVID-19 than in uninfected individuals. The levels of antibodies in individuals with different AB0 blood types also varied: patients with O and B blood types had higher anti-Tn levels than patients with A and AB blood types [19]. According to epidemiological data, patients in the first group get infected with COVID-19 less frequently and mostly have mild disease than those with the second and fourth blood groups. The involvement of anti-Tn in antiviral immunity also confirms the presence of a strong correlation between the levels of these antibodies and antibodies to the SARS-CoV-2 S-protein. 

### 7.3. Anti-Glycan Antibodies in COVID-19: Own Data

A detailed description of the study of anti-glycan antibodies in patients with COVID-19, including the Sections “Materials and methods” and “Results”, is presented in the “Supplementary” file.

The mentioned PGA contains 695 glycoligands (mammalian glycans, their fragments, and O-polysaccharides of pathogenic and conditionally pathogenic bacteria); it was used to compare antibody profiles (IgM and IgG separately) of patients who underwent COVID-19 of various severity and relatively healthy donors whose serum has been collected before 2019. The levels of antibodies to some glycans were significantly higher than to the others. (In particular, to the carbohydrate part of glycosphingolipid Gb5, which is expressed on the cells of the immune system [147]; to the -Galβ1-4Glc motif, which is the inner core of all glycosphingolipids; to chito-oligosaccharides, (GlcNAcβ1-4)_n_; to O-phosphorylated lactosamine and O-polysaccharides containing phosphate residues from *Escherichia coli*, *Shigella flexneri*, and *Pseudomonas aeruginosa*). For another group of glycans (rhamnose, melibiose, a trisaccharide fragment of the Forssman antigen, and glucosaminyl muramylpeptide, a peptidoglycan fragment of the bacterial cell wall), the reduced levels of antibodies compared to donors were detected. A wide variety of antibodies to the array of about 200 bacterial polysaccharides was observed; however, we did not analyze them in their appearance in relation to coronavirus disease, since these mostly adaptive antibodies reflect an individual’s recent history of interaction with pathogenic bacteria.

**Figure 1:**
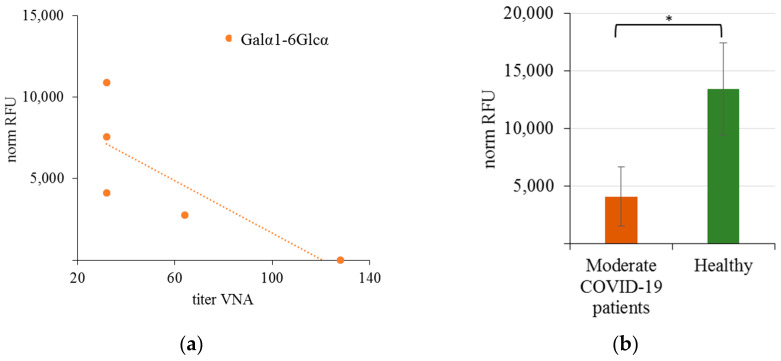
(**a**) Correlation between the level (as norm RFU value) of binding of IgG antibodies to Galα1-6Glcα, and VNA in patients who underwent moderate COVID-19; r_s_ values exceeding the absolute value of 0.6 with *p* < 0.05 were taken into account; (**b**) binding activity of IgG to Galα1-6Glcα in moderate COVID-19 patients and in healthy donors (* *p* < 0.05).

**Figure 2:**
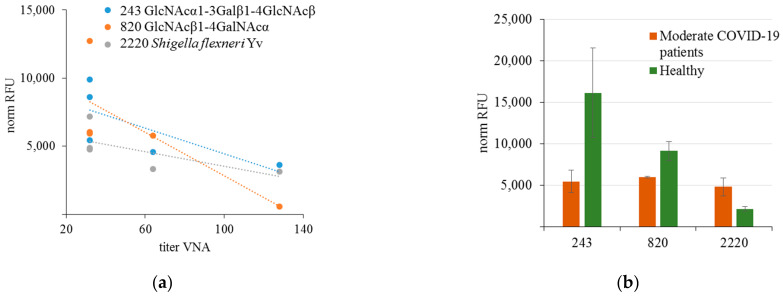
(**a**) Correlation between the level (as RFU value) of binding of IgM antibodies to glycans, and VNA in patients who underwent the moderate form of COVID-19; r_s_ values exceeding the absolute value of 0.6 with *p* < 0.05 were taken into account; (**b**) binding activity of IgM to glycans 243, 820 and 2220 in moderate COVID-19 patients and in healthy donors (*p* < 0.05 in all cases).

As shown above, the number of antibodies species for which different AGA levels were found in infected patients and healthy controls is quite large. The observed differences were not dramatic for any specificities, and there was no obvious structural similarity in the detected glycans. We expected to find more definite links between AGA and coronavirus disease when analyzing the VNA of blood antibodies. Therefore, for the same cohorts, we measured the VNA of blood serum in Vero C1008 cell culture against SARS-CoV-2 coronavirus (variant B). Correlation analysis of antibody binding to glycans was performed separately in three cohorts: (1) patients with mild disease, (2) COVID-19 patients with moderate disease, and (3) healthy donors whose blood was collected before 2019. No positive or negative correlations were found for patients in the second cohort, while for the first one, we have identified negative correlations (according to Spearmen’s rank) of moderate strength (r > 0.6), both for IgG (Figure 1) and IgM (Figure 2).

For Galα1-6Glcα and GlcNAcα1-3Galβ1-4GlcNAcβ, the levels of antibodies in infected patients were lower than in healthy controls; the correlation was opposite for GlcNAcβ1-4GalNAcα. It should be noted that reciprocal correlations between AGA levels and serum VNA were found for one AGA IgG and three IgM antibodies. ADE-phenomenon studies have mainly focused on IgG, but the involvement of antibodies of other classes has also been proven [2]. Given that the described levels of some natural AGA in COVID-19 patients and convalescents (particularly anti-Tn antibodies, anti-A, and anti-B alloantibodies) [19,145] were significantly lower than in healthy subjects, their potential VNA can be assumed; however, this requires further proof.

The levels of antibodies to three glycans (Figure 2) correlated with VNA in convalescents after moderate COVID-19 infection; none of these glycans can be synthesized by a normal set of human cell glycosyltransferases. Possibly, AGAs do not recognize these glycans *per se* but rather the spatial epitopes formed by sugar residues from different carbohydrate chains or adjacent molecules of different nature (molecular patterns). Peculiarities of AGA binding to glycans on PGA were demonstrated earlier [148], which allowed us to illustrate differences in the specificity of affinity isolated anti-LeC and their binding to a range of closely related structures. Since one of the properties of natural AGA is their polyreactivity and ability to bind PAMPs, their role in the antiviral response in COVID-19 and ADE is quite probable. However, based on our study, we cannot conclude whether these antibodies are natural or adaptive; this is a limitation of the research, since the AGA profile has not been examined before the disease. Therefore, there is a need for further studies with more representative cohorts and experimental models that can prove the binding of affinity isolated AGA to SARS-CoV-2 and identify their carbohydrate epitopes, peptide, or glycopeptide mimotopes.

## 8. Conclusions

The issue of ADE is one of the most enigmatic topics in immunology. The main paradoxical questions that arise and that have not been answered so far, despite the nearly 60-year history of research on this phenomenon, are discussed in the first part of the manuscript. The key players in ADE are antibodies. Throughout the research history of the phenomenon, natural antibodies have never been the subject of relevant investigation. Moreover, in the context of ADE, the assignment of the term “preexisting antibodies” to adaptive anti-virus immunoglobulins needs to be reconsidered, since true preexisting antibodies are those that are present in the human blood before contact with foreign antigens, i.e., natural antibodies. Natural antibodies bind to patterns of foreignness and mediate the antiviral response, which plays a leading role in the first stage of infection. The lower incidence of COVID-19 in individuals with type 0(I) blood group due to alloantibodies is one (but not the only) strong argument supporting the role of natural anti-glycan antibodies in the early antiviral response. Given a high degree of glycosylation of viral proteins, anti-glycan antibodies are involved both in the immune response to viral aggression and in the development of ADE; both involvements are supported by the evidence demonstrated in this review. However, the number of such studies is disproportionately small, while most studies of antiviral immunity are addressing adaptive antibodies to viral epitopes of protein nature. The denial of the role of anti-glycan antibodies in antiviral immunity is logically based on the almost identical glycosylation of the virus and the human cell. But this statement cannot be considered as absolutely true, since the structure of the carbohydrate chain synthesized in the cell (and, accordingly, its antigenicity) also depends on the protein chain on which it is assembled in the Golgi apparatus, and viral proteins obviously differ from human ones. It is very likely that there are such AGA that recognize a complex (possibly spatial) epitope composed of a glycan fragment and a peptide (or lipid) region. It is this organization of the epitope that can explain the specificity of antibodies of mysterious epitope specificity, the level of which reciprocally correlates with the VNA of COVID-19 convalescent’s serum; this assumption justifies the need for further research in this area.

The findings on the glycosylation of DENV proteins and the proven role of carbohydrate-protein interactions in virus attachment and penetration into cells are the additional arguments in favor of further studies of the role of AGA in the development of ADE phenomenon. No evidence for the involvement of AGA in the pathogenesis of ADE has been reported to date. However, it is clear that AGAs have the potential to bind to DENV glycans, and the study of this aspect may contribute to the understanding of the pathogenesis of ADE which develops in dengue shock syndrome.

The presented data indicate that many questions related to ADE are still to be answered, and therefore, further study of this phenomenon is required, where special attention should be paid to anti-glycan antibodies since they are true pre-existing antibodies by means of which the immune system immediately comes into contact with the virus.

## Data Availability

Data are available upon reasonable request from the corresponding author.

## Supplementary Materials

The following supporting information can be downloaded at: https://www.mdpi.com/article/10.3390/v15071584/s1; Supplementary I [149]: the study of anti-glycan antibodies in patients with COVID-19, including the material, methods and results; Supplementary II: the list of ligands.

## Abbreviations

Angiotensin-converting enzyme 2	ACE2
Antibody-dependent enhancement	ADE
Anti-glycan antibodies	AGA
already existing antibodies	AEAbs
Complement receptors	CRs
Fc-receptors	FcRs
Receptor-binding domain	RBD
Pathogen-associated molecular patterns	PAMPs
Printed glycan array	PGA
Virus-neutralizing activity	VNA
Carbohydrate abbreviations	
Galβ1-4GlcNAcβ	LacNAc
GalNAcβ1-4GlcNAcβ	LDN
Galβ1-3(Fucα1-4)GlcNAcβ	Lewis X, LeX
Galβ1-4GlcNAcβ1-3Galβ1-4GlcNAcβ1-3Galβ1-4GlcNAcβ1-3Galβ1-4Glc	LNnO
Fucα1-2Galβ1-3GlcNAcβ	H type 1
GalNAcα1-3GalNAcβ1-3Galα1-4Galβ1-4Glcβ	Forssman antigen, Fs
GalNAcα	Tn
(GlcNAcβ1-4)_n_	chito-oligosaccharides
