# Supplementary material for: Antibody-Dependent Enhancement with a Focus on SARS-CoV-2 and Anti-Glycan Antibodies
Source: Viruses. 2023 Jul 20;15(7):1584. doi: 10.3390/v15071584 (PMC10384250; doi:10.3390/v15071584)
Supplement: Supplementary file 1 [file viruses-15-01584-s001.zip › viruses-2491227-Supplementary SI.pdf]

Supplementary SI

1. Materials and Methods

1.1. Patients

The study included 15 women who underwent COVID-19 between March 25 and May 20, 2020, and were treated at the Kulakov National Medical Research Center for Obstetrics, Gynecology and Perinatology of the Ministry of Health of the Russian Federation. All patients signed an informed consent to participate in the study. The study was approved by the Ethics Committee of the Kulakov National Medical Research Center for Obstetrics, Gynecology and Perinatology of the Ministry of Health of the Russian Federation (act N4 of April 23, 2020).

Inclusion criteria: age over 18 years, female, signed informed consent, COVID-19 confirmed using the "SARS-CoV-2/SARS-CoV" kit for the detection of SARS-CoV-2 and similar SARS-CoV RNA ("DNA-Technology TS", Russia; RU 2020/9948), which identifies three targets: conservative site of the SARS-CoV E-gene; specific site of the SARS-CoV-2 E-gene; specific site of the SARS-CoV-2 N-gene. Analytical sensitivity of the test is $5x10^2$ copies/ml.

Non-inclusion criteria: pregnancy or lactation, cancer; history of organ transplantation or hemotransfusion; acute infectious diseases other than COVID-19.

Exclusion criteria: need for surgical intervention during COVID-19, patient's refusal to continue participation in the study.

Patients were stratified into groups based on the severity of COVID-19: group 1 consisted of women who had had mild or asymptomatic COVID-19 form (n=10); group 2 included women who moderate COVID-19 form (n=5). Diagnosis of the moderate disease: fever >38°C, respiration rate >22 rpm, dyspnea, lung tissue lesions at CT (volume of lesions is minimal or medium, CT-1 or CT-2). In the absence of these signs the diagnosis of a mild disease of COVID-19 was made. Also, the blood of the donors later provided by the Biobank was studied for comparison (healthy donors).

1.2. Blood serum sampling

Peripheral blood sampling was performed in patients between 6-14 weeks after the onset of the disease. Fasting blood sampling was performed and serum samples were collected in S-Monovette® vacutainer tubes with a white lid and clot activator (4.9 ml, L×Ø = 90 x 13 mm). One hour after blood collection, samples were centrifuged for 10 min at 2000×g, and stored at -80°C until the antibody analysis was performed.

1.3. Determination of specific antibodies to SARS-Cov-2 using commercial ELISA kits

SARS-CoV-2 antibodies were detected using a reagent kit for enzyme immunoassay for total antibodies (IgM, IgA, IgG) to recombinant SARS-CoV-2 antigens in blood serum (plasma) (XEMA, Russia) according to the manufacturer's instructions. Based on the data obtained, the relative activity of antibodies was defined by calculation of the so-called positivity index, which is the ratio of the optical density of the patient's sample to the threshold value defined in the system. The cut-off value was calculated by multiplying the average optical density of the positive controls (positive samples containing antibodies) by A coefficient (this coefficient is specified in the kit's analytical data sheet).

1.4. The analysis of virus neutralizing activity

To study the virus neutralizing activity (VNA) of the blood sera of convalescents the Vero C1008 cell culture (cell monolayer in a flask) and SARS-CoV-2 coronavirus (variant B) based on Vero C1008 cell culture was used. Vero C1008 cells were cultured in culture flasks with a 225 cm2 working surface area using medium 199 with Hanks salts, supplemented with 7.5% fetal bovine serum (FBS), glutamine, glucose, and gentamicin.

The working dilution of the virus was prepared in Hanks' solution with 2% FBS and 100 U/ml of antibiotics, by 10-fold dilutions of the initial virus-containing suspension until a final concentration of the virus in the sample of 200 BAU ml^{-1}.

Dilutions of the patients' test sera were prepared using saline with the addition of antibiotics (streptomycin sulfate and benzylpenicillin sodium salt at 100 units/ml in two-fold increments). The initial dilution of the serum was 1:4; the final dilution was 1:256. As a positive control, we used the convalescent's blood serum demonstrating VNA to the SARS-CoV-2 virus at a dilution of 1:25. Fetal calf serum at a dilution of 1:4 was used as a negative control.

Blood serum VNA of convalescents was determined in the neutralization reaction by suppression of negative colony formation caused by SARS-CoV-2 virus in a one-day monolayer of Vero C1008 cells under agar overlay.

A mixture of equal volumes of serum and working dilution of SARS-CoV-2 virus culture was incubated for 60 min at 36.5 to 37.5oC; then 0.5 ml of mixture was applied to a monolayer of Vero C1008 cells. After adsorption of the antigen-antibody complex on the cells for 60 min at 37oC, the inoculum was decanted, then the primary agar overlay for SARS-CoV-2 virus was applied, and further incubation of the monolayer at the above temperature was performed for two days. Next, a secondary staining agar overlay with 0.1% neutral red was applied to the infected cell monolayer, and incubated for 24 hours at 36.5 to 37.5^{o}C; afterwards, the negative colonies were counted in flasks. The VNA titer was defined as the highest dilution of serum that caused a 50% reduction in the number of plaques (BAU), compared to the average viral control.

1.5. Profiling of serum antiglycan antibodies with a printed glycan array (PGA)

1.5.1. Glycoarray printing and quality control

A library containing 695 ligands consisting of oligosaccharides - terminal and core parts of mammalian cell glycoconjugates, O-polysaccharides of pathogenic and opportunistic bacteria (courtesy of Yu. Knirel, N.D. Zelinsky Institute of Organic Chemistry, Russia) was printed on NHS-activated glass as an array (Semiotik LLC, Russia). All ligands had high purity (at least 95%). A complete list of ligands is provided in Appendix.

Before printing, the ligands were dissolved in a buffer solution (300 mM phosphate buffer, pH 8.5, containing 0.001% Tween 20 (ICN, USA) to achieve a concentration of 20 μM for oligosaccharides and 2 mg/ml for O-polysaccharides. Printing was performed on activated H slides (Schott Nexterion, Germany) using a SciFlex ARRAYER S5 contact-free robot (Scienion, Germany), droplet volume ~900 pl, relative humidity at 50%. Each ligand was printed at 6 replicates. After printing the ligands, the chips were incubated for 1 h at 70% relative humidity; afterwards, the remaining active groups were blocked with blocking buffer (25 mM ethanolamine (SigmaAldrich, USA) in 100 mM borate buffered saline, pH 8.5, containing 0.1% Tween 20) for 1 h at room temperature.

The glycoarrays underwent a multistep quality control, as follows:

1. Application of the ligand solution to the activated slide was controlled using robot software. If necessary, the ligands were additionally printed.

2. Visual control of the chip after processing with standard plasma.

3. Assessment of intra- and interslide correlation. Glycoarrays with correlations greater than 0.8 were considered to have passed quality control.

1.5.2. Profiling of glycan-binding serum antibodies by PGA

The PGA were incubated for 15 min in 0.1 M isotonic phosphate buffer saline (PBS) containing 0.1% Tween 20 (PBS-0.1%). They were then covered with the studied serum, 15-fold diluted in PBS-0.1% containing 3% BSA (1 chip, 1 sample) and incubated on a shaker at relative humidity of 80% for 1 h. The glycoarrays were then washed with PBS-0.05%, and the goat antibodies, recognizing human IgG and IgM labeled with Alexa647

and Cy3 (Jackson Immuno Research, USA), respectively, and diluted at 1:2500 in PBS-0.1% were applied onto array surface. After incubation on a shaker at relative humidity of 80% for 1 h, the PGA were washed first with PBS-0.05%, then with double-distilled water.

Antibody levels were measured using an Innoscan 710 confocal scanner with a resolution of 10 μm in relative fluorescence units (RFU). The data were processed using ScanArray Express 3.0 software using fixed circles with a diameter of 80 μm. Signals that exceeded the signals from the surface, which did not contain the ligand by 10 times, were considered as significant.

1.6. Statistical analysis

Primary data processing was performed using Microsoft Office Excel: median signals and median absolute deviation (MAD) by repeats of each ligand were calculated. Statistical analysis: chip data normalization by quantile normalization over the entire data set; Wilcoxon-Mann-Whitney test (WMW-test); χ^2-test; t-test, and Spearman correlation analysis was carried out using R, MedCalc, and Statistica 10.0 software package.

2. Results

2.1. Characteristics of the study groups

Medical history and clinical data of the patients with COVID-19 are presented in Table S1.

Table S1. Clinical and laboratory data of the patients from Groups 1 and 2.

Parameters	Group 1 (n=10)	Group 2 (n=5)	p-level
Age, years*	41.1±9,6	43.4±9.6	0.669
BMI, kg/m2*	24.3±4.9	24.4±3.2	0.962
Smoking**	1 (10%)	1 (5%)	0.591
Blood group A(II)	8 (80%)	3 (60%)	0.409
Rh(-)	0	2 (40%)	**0.032**
Chronic tonsillitis	3 (30%)	1 (20%)	0.680
Chronic rhinitis	1 (10%)	2 (40%)	0.171
Allergic background	0	2 (40%)	**0,032**
History of pediatric infections	4 (40%)	1 (20%)	0.439

[1] * the data are presented as averages ± standard deviation, t-test;
** the data are presented as absolute numbers and %, χ^2-test;
BMI – body mass index; Rh(-) – rhesus-negative blood.

8 healthy donors whose blood was sampled and stored before 2019 were included in the study.

The average age of the patients in both groups was 41.8±9.3 years (31 to 56 years), the average BMI was 24.3±4.3 kg/m2 (19.8 to 35.8 kg/m2). The majority of patients in both study groups had A(II) blood group (11 persons, 73.3%). There was no history of a complicated somatic, allergic or infectious disease in the patient's records in both study groups. Most often chronic diseases (in remission or well-controlled) were: chronic tonsillitis – 26.6 %; chronic rhinitis – 20%; hypertension – 6,6%. A history of allergic events was documented in two patients (13.3%). In general, the observed cohort of patients had a low incidence of past viral infections. Only three of 15 patients (20%) had a history of childhood infections (varicella, mumps, and rubella).

2.2. Identification of virus neutralizing activity

The serum VNA in Vero C 1008 virus-infected cell culture was determined at 6-14 weeks after disease onset (variant B).

Table S2 shows the titer of the patient's serum, which revealed suppression of negative colonies formed by SARS-CoV-2 virus by 50% or more, compared to the control.

Table S2. . Results of virus neutralizing activity of the patients' blood serum.

Group	Serum code	VNA titer	Weeks from onset of disease
1	ZBN188	1:32	10
1	ZBN197	1:32	13
1	ZBN198	1:32	7
1	ZBN199	1:16	10
1	ZBN196	1:4	8
1	ZBN191	1:32	6
1	ZBN189	1:32	13
1	ZBN192	1:64	10
1	ZBN195	1:32	14
2	ZBN850	1:128	6
2	ZBN194	1:32	13
2	ZBN193	1:64	13
2	ZBN190	1:32	12
2	ZBN187	1:32	12
healthy donors	BRFAMKR79	1:8	-
healthy donors	BRMESZB12	1:8	-
healthy donors	BRFAMKR85	1:8	-
healthy donors	BRFAMKR62	1:8	-
healthy donors	BRMESZB17	1:8	-
healthy donors	BRFAMKR44	< 1:4	-
healthy donors	BRSPUZB105	< 1:4	-
healthy donors	BRFAMKR69	< 1:4	-

[1] Group 1 – mild or asymptomatic form, Group 2 – moderate form

The results presented in Table S2 indicate the presence of virus neutralizing activity to SARS-CoV-2 in 83% of the serum samples of the convalescents. VNA were detected in the titers of 1:8 in 25% of samples; 1:16 in 4%; 1:32 in 42%; 1:64 in 8%; and 1:128 in 4%. In 17% of serum samples, the VNA titer to SARS-CoV-2 was less than 1:4. No significant differences in VNA titers between the two compared groups were found (p=0.1034, t-test).

2.3. Detection of antibodies to SARS-Cov-2 S-protein

A commercial Xema LLC kit was used to detect high level of antibodies to SARS-CoV-2 protein in patients' serum, which was significantly different from the antibody level of healthy donors (Figure S1).

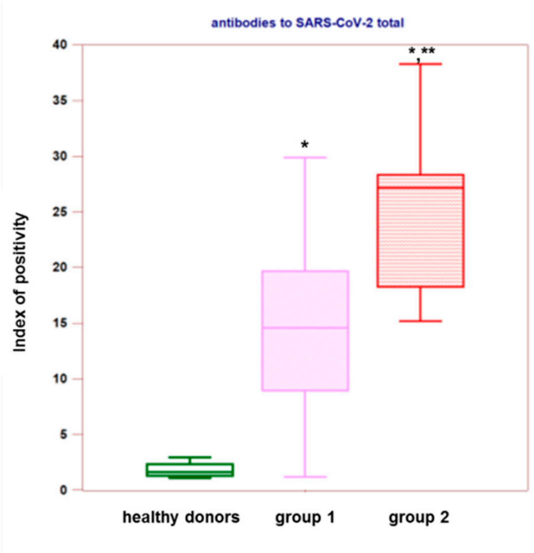

Figure S1. Level of antibodies to SARS-CoV-2 S-protein in healthy donors and in COVID-19 patients. * - comparison to healthy donors; ** - comparison of Group 1 with Group 2.

The median antibody positivity index for SARS-CoV-2 was 1.67 (1.34-2.38) in healthy donors; 14.61 (8.99-19.69) in Group 1 patients (p=0.009, for healthy donors); and 27.18 (18.27-28.32) in Group 2 patients (p=0.002, for healthy donors). Significant differences in positivity index were found between Group 1 and Group 2 patients (p=0.04).

2.4. Studying the antibody repertoire with PGA

In order to find significant antiglycan antibodies (AGA), the levels (expressed as RFU) of their binding to the array glycans were compared in healthy donors *vs* patients. The data below include only the changes in the levels of AGA (both IgG and IgM), which turned out to be opposite in the healthy donors versus Covid-19 patients. In some cases, AGA levels were higher in the convalescent group (upper part of the heat maps below), and in other cases they were higher in the healthy donors group (lower part of the heat maps).

2.4.1. Comparison between the AGA profiles in Group 1 and in the healthy donor group

Heat map analysis shows that in AGA, the IgG profile of patients with mild COVID-19, the antibodies to *Escherichia coli* O51 and *Salmonella enterica* O57 O-polysaccharides (O-PS), which have a similar repeating unit [149] , and to the amino acid derivatives of lactose, were prevailing (Figure S2A). Along with a high representation of AGA IgG of the above specificities, the profile of Group 1 shows a low representation of antibodies to a number of O-polysaccharides containing the GlcNAcβ1-2Rhaα1-2Rhaα1-3 fragment, as well as to Galα1-4- or Galα1-6-terminated glycans.

The AGA IgM profile (Figure S2B) is characterized by: 1) high levels of antibodies to a number of bacterial O-polysaccharides with different structural composition, and to a significant number of sulfated oligosaccharides; 2) low levels of antibodies to xeno-disaccharide (Galα1-3Galβ) and to O-PS of bacteria serotypes, other than those of the AGA IgG profile.

It should be noted that antibodies to LacdiNAc (GalNAcβ1-4GlcNAcβ) sulfated at various positions of the terminal GalNAcβ residue, demonstrated the highest binding among the AGA of both classes.

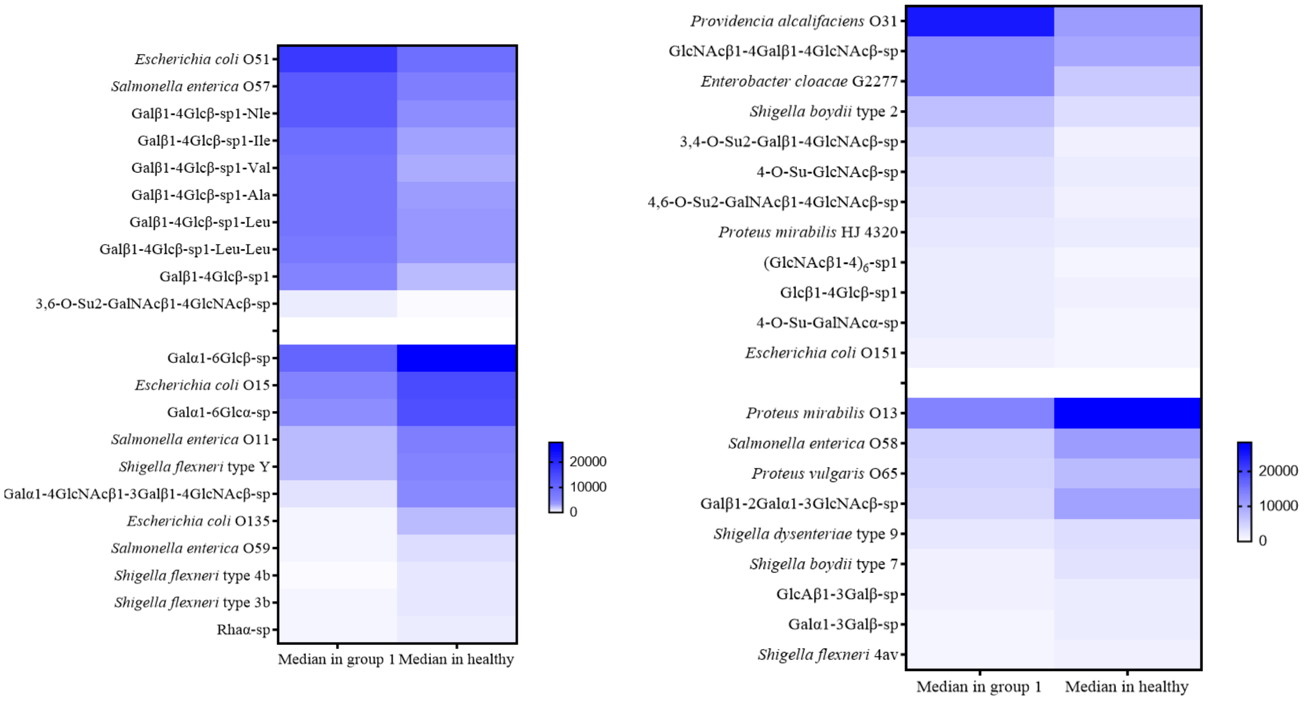

(**a**) (**b**)

Figure S2. Heat maps of interactions between antibodies and glycans for Group 1 patients and healthy donors ($p<0.05$). (**a**) IgG; (**b**) IgM. The color scale reflects the change in median fluorescence intensity in the respective groups. *Hereinafter, all monosaccharides are D pyranoses unless otherwise indicated. sp - aminoethyl or aminopropyl spacer, sp1 - glycyl spacer. A complete list of structures is provided in Appendix (List of ligands).

2.4.2. Comparison of AGA profiles in Group 2 and healthy donors

The profile of significant AGA IgG that distinguishes Group 2 from healthy donors is narrower than that described for Group 1 (Figure S3A). The AGA IgG profile in moderate COVID-19 patients also showed notable difference, compared to healthy donors, in the levels of antibodies to a number of bacterial O-polysaccharides: significantly higher levels of antibodies to the carbohydrate component of glycosphingolipid Gb5; and significantly lower levels to the carbohydrate component of glycosphingolipid Fs, to the phosphorylated polysaccharide of *Proteus penneri* 31 (and other phosphorylated polysaccharides), and to a component of the bacterial cell wall polysaccharide GlcNAcβ-Mur. AGA IgG level to the disaccharide Galα1-6Glcα was also higher in healthy donors.

The AGA IgM profile was broader than the AGA IgG profile in Group 2. Antibody levels were higher to phosphorylated polysaccharides of *Pseudomonas aeruginosa* O14, *Escherichia coli* O151, and *Proteus vulgaris* OX19, to the bacterial monosaccharide αKDO-5-phosphate (Figure S3B). Antibodies to O-polysaccharide of *Enterobacter cloacae* (strain G2277) were also found when comparing the AGA IgM profiles of the healthy donors and Group 1. Group 2 had lower AGA IgM concentrations to a number of non-natural oligosaccharides, and to phosphorylated O-polysaccharide (O-SP), e.g. from of *Shigella flexneri* 4av. It should be noted that of the 695 ligands presented on the chip, only 24 were phosphorylated (19 O-SP and 5 mono-/oligosaccharides).

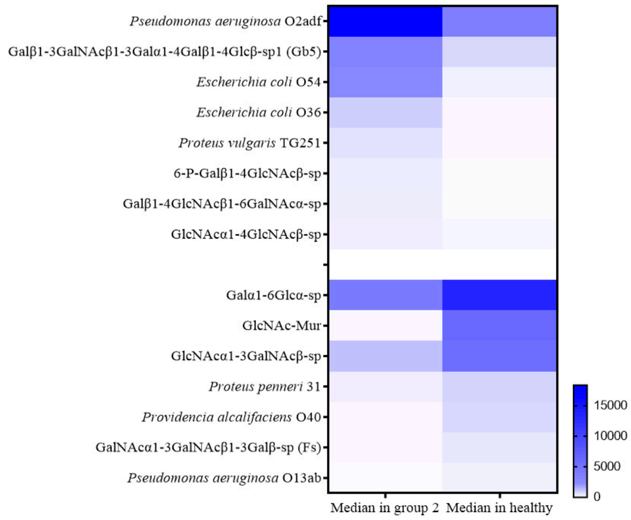

(**a**)

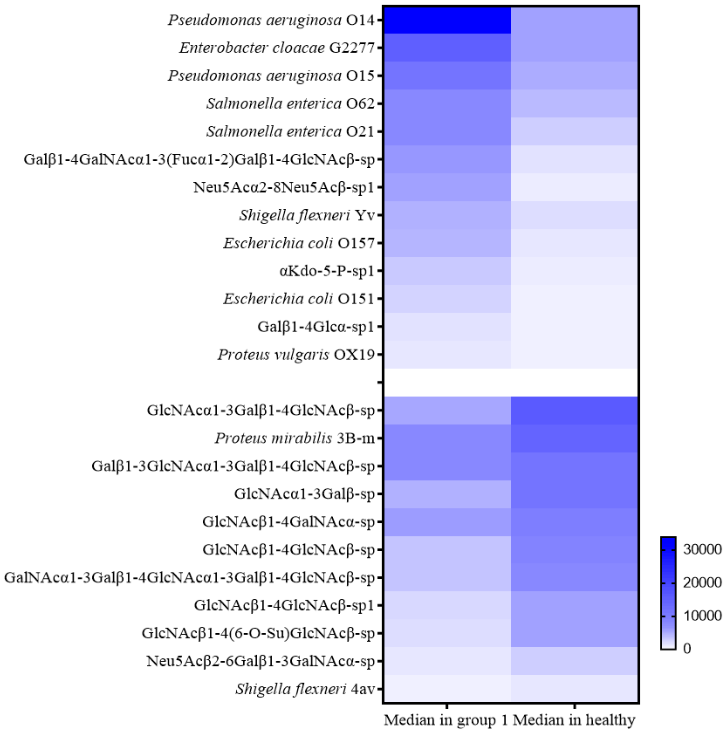

(**b**)

Figure S3. Heat maps of interactions between antibodies and glycans in Group 2 and healthy donors ($p<0.05$). (**a**) – IgG; (**b**) – IgM. The color scale reflects the change in the median fluorescence intensity. P – phosphate, Mur – muramoyl.A complete list of structures is provided in Appendix (List of ligands).

2.4.3. Comparison of AGA profiles in Group 1 and Group 2

The studied AGA (IgM and IgG) in Group 1 and Group 2 (Figure S4A,B) significantly (up to 50%) overlapped with the specificities found in the previous comparisons.

However, the antibodies that were specific only for this comparison were also found. In particular, levels of AGA IgG to a number of O-PS (including phosphorylated *Salmonella enterica* O47-deAc and *Escherichia coli* O151), to the component of a bacterial cell wall GMDP-Lys, and to non-natural *α*-N-acetylactosamine and GalNAc*α*1-4GalNAc*α* (Figure S4A), was higher in Group 1, and levels of AGA IgG to glycans with a core GalNAc*α* residue and polysaccharides of *Cronobacter sakazakii* G2356 O2 and *Escherichia coli* O54 with common GalA*α*1-2Rha*α* component was higher in Group 2. IgM to GlcNAcβ and GlcNAc*α*- terminated oligosaccharides had higher signals in Group 1, and IgM to GalNAcβ glycan and a number of O-PS had higher signals in Group 2 (Figure S4B).

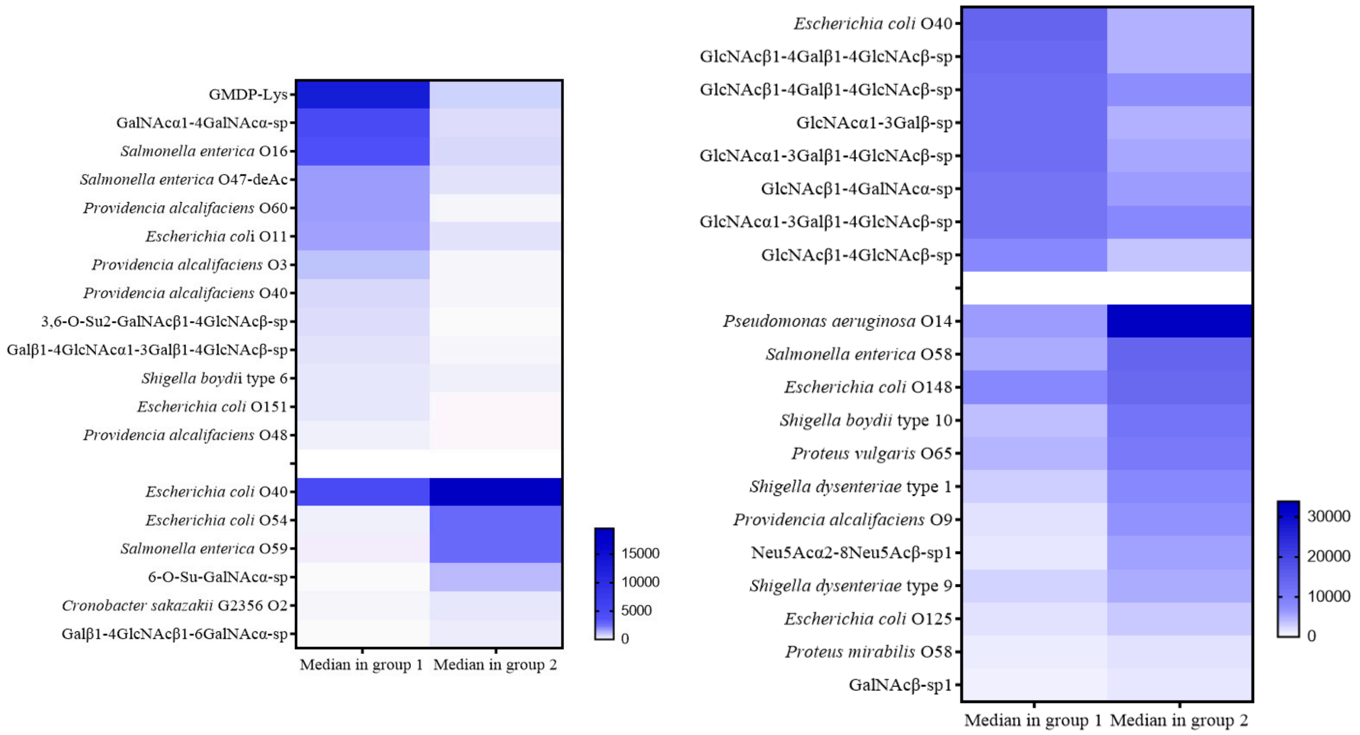

(**a**) (**b**)

Figure S4. Heat maps of interactions between antibodies and glycans in Group 1 and Group 2 patients (p<0.05). (**a**) IgG; (**b**) IgM. The color scale reflects the median fluorescence intensity.

2.5. Correlations between the levels of IgM and IgG AGA, and the virus neutralizing activity of blood serum

The correlation between VNA and AGA levels was determined by correlation analysis (Spearman's rank correlation coefficient) separately for Groups 1 and 2. A significant number of direct and inverse correlations were found both groups in Group1 and Group 2) (Figures S5 and S6).

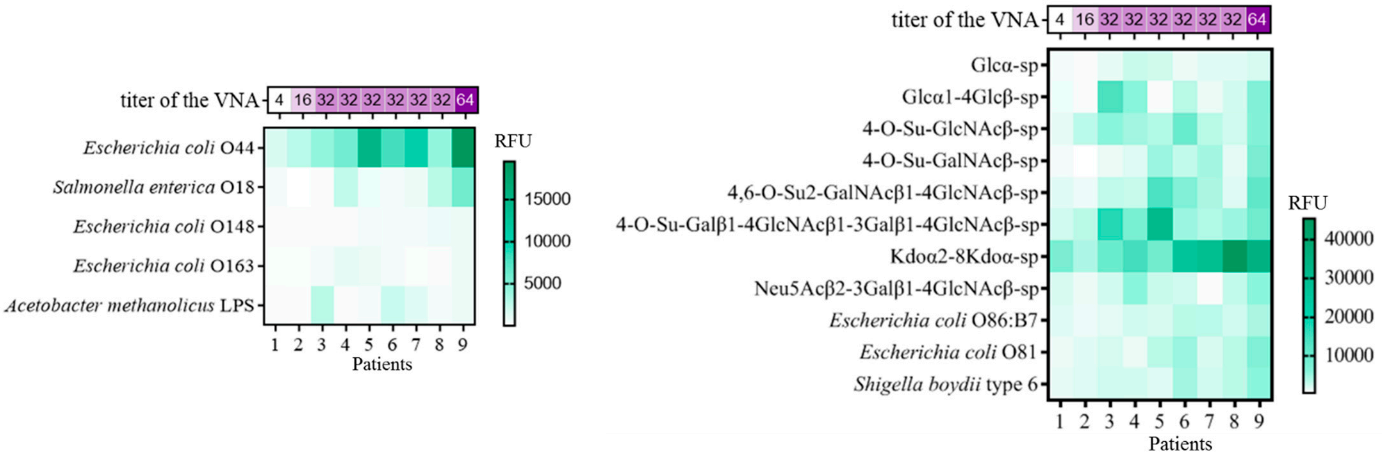

(**a**) (**b**)

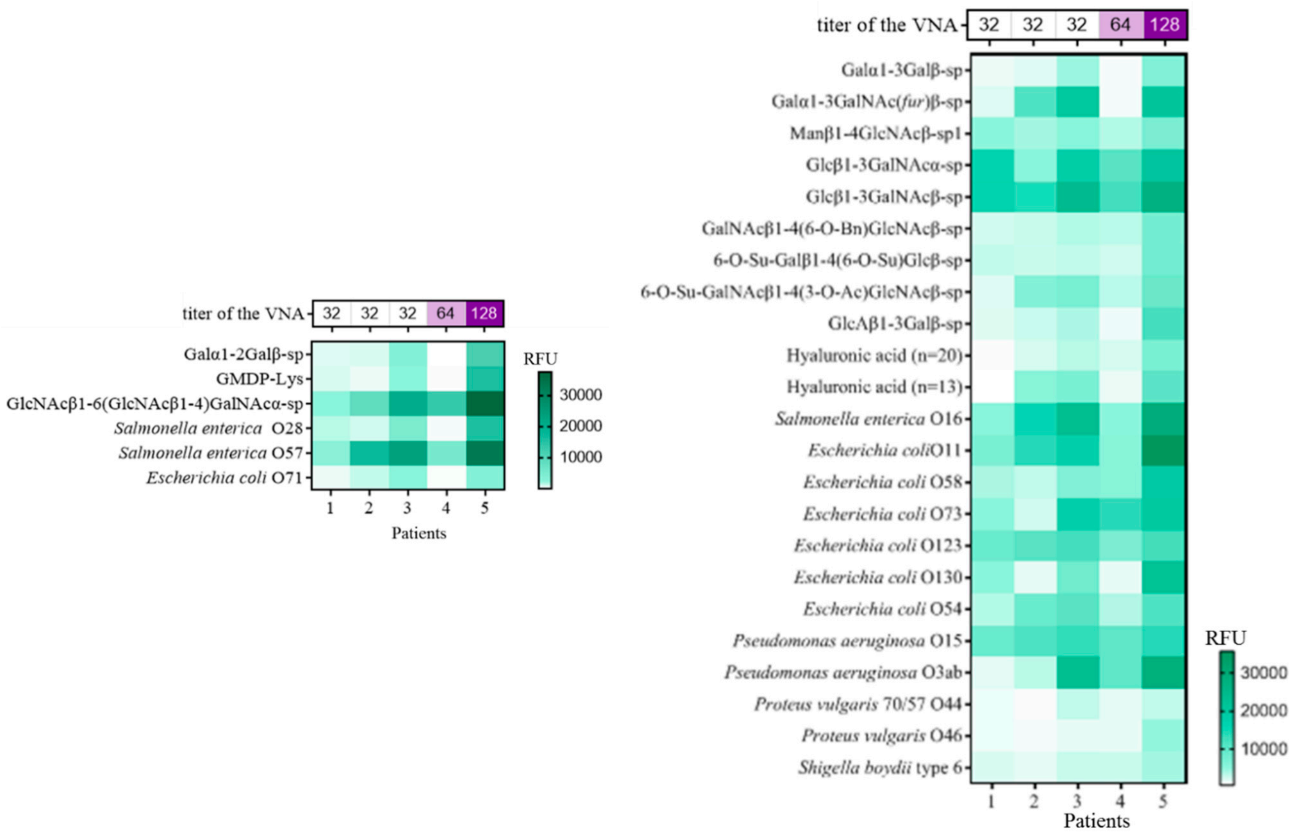

(**c**) (**d**)

Figure S5. Direct correlations between the levels of antiglycan antibodies (the levels are expressed as RFU) and the serum titer for VNA. The glyco-ligands targeted by AGA are shown on the left. The heat map shows the RFU of the antiglycan antibodies for each patient included in the study. Spearman's rank correlation coefficients (r_s) with an absolute value greater than 0.6 and $p<0.05$ were taken for analysis. (**a**) IgG in Group 1; (**b**) IgM in Group 1; (**c**) IgG in Group 2; (**d**) IgM in Group 2. RFU – relative fluorescence units.

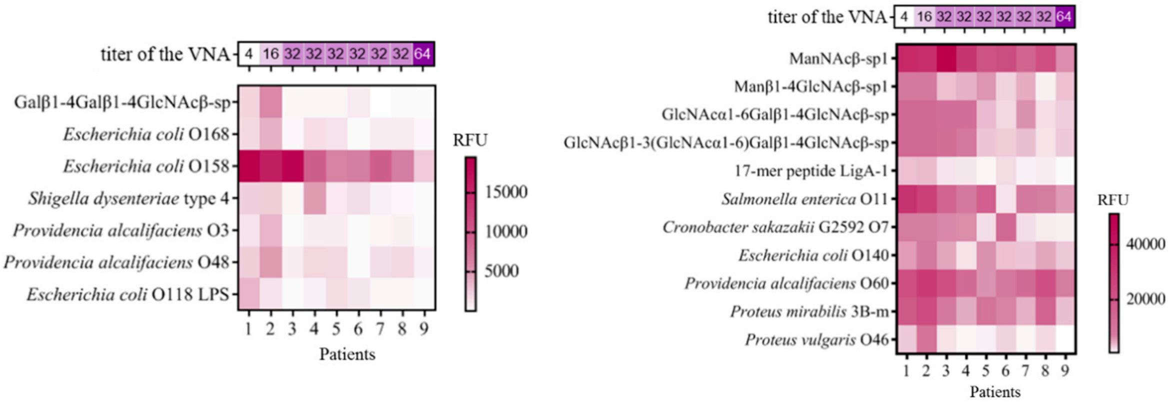

(**a**) (**b**)

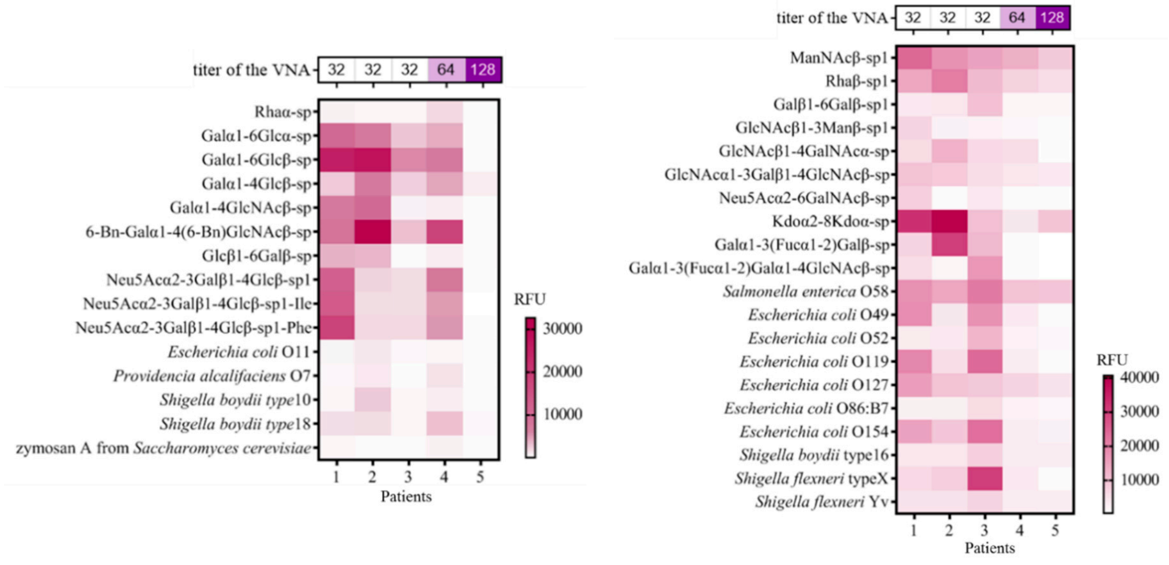

(**c**) (**d**)

Figure S6. Reciprocal correlations between the levels of antiglycan antibodies and the serum titer for VNA. Spearman's rank correlation coefficients > 0.6 with p< 0.05. (**a**) IgG in Group 1; (**b**) IgM in Group 1; (**c**) IgG in Group 2; (**d**) IgM in Group 2. RFU – relative fluorescence units.

In Group 1, negative correlations with VNA were found for IgG to O-PS of several bacteria, including *Echerichia coli* O168, O158 and O118; to *Shigella boydii* type 14 and fragment of its repeat unit - trisaccharide Galβ1-4Galβ1-4GlcNAcβ. Also negative correlations were noted for antibodies to O-PS *Providencia alcalifaciens* O3 and O48, which were found in a comparison of AGA IgG profiles of Group 1 and Group 2, with a higher binding activity observed in Group 1. The VNA level negatively correlated with IgM to bacterial O-polysaccharides, including those belonging to the families *Enterobacteriaceae* (*Proteus, Echerichia*), *Morganellaceae* (*Providencia alcalifaciens* O60); to the fragments of N-linked glycoproteins chain Manβ1-4GlcNAcβ; and to ManNAcβ and GlcNAcα-terminated saccharides that are not present in mammals.

Negative correlations with VNA were observed for antibodies of both classes to polysaccharides of various *Escherichia* and *Shigella* strains. Negative correlation for IgM to *Escherichia coli* O86:B7 and its B trisaccharide, a structural component of its repeating unit, deserves special attention. In contrast, a positive correlation was observed for IgM to this polysaccharide in Group 1, r_s=0.73; p=0.025. A negative correlation was found for IgM to ManNAcβ and GlcNAcα- terminated epitope (similar to Group 1, see above), as well as to Rhaβ (negatively correlating IgG to α-anomer) and to Kdoα-2-8Kdoα, components of the core part of bacterial polysaccharides. In Group 1, IgM to Kdoα-2-8Kdoα had a positive correlation with VNA (r_s=0.75; p=0.019). We also found in Group 2 a negative correlation of VNA with IgG to to Galα1-6 and Galα1-4-terminated epitopes and amino acid derivatives of 3'-sialillactose.

To identify antibodies associated with ADE, only strong and medium reciprocal correlations for both IgG and IgM were considered, while the level of antibodies in the groups of patients and healthy control should have been significantly different. Such antibodies were found only in Group 2 (moderate disease); they belonged to both classes of immunoglobulins – IgM and IgG (Figure S7).

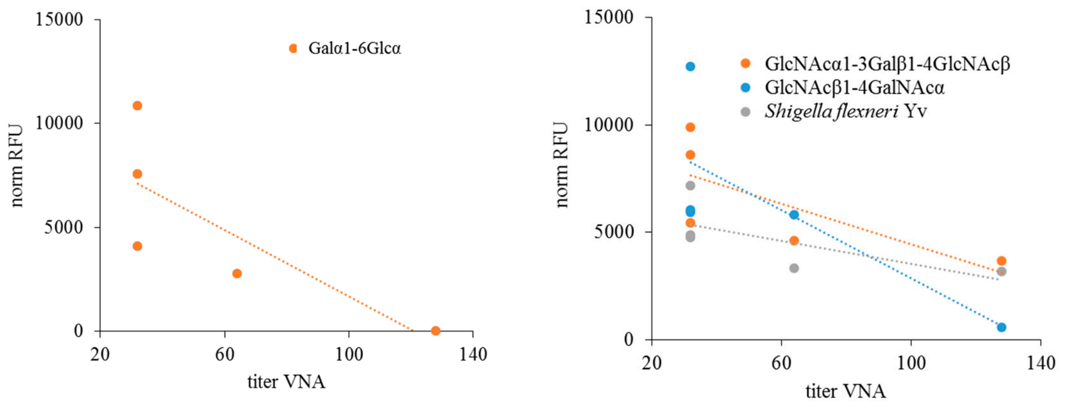

(**a**) (**b**)

Figure S7. Correlation between the levels of (**a**) IgG and (**b**) IgM antibodies (expressed in RFU) to glycans and VNA in moderate COVID-19 patients (Group 2); only r values greater than 0.6 with significant difference between the patients from Group 2 and healthy donors ($p<0.05$) were considered.
