# Supplementary material for: Antibody-Dependent Enhancement with a Focus on SARS-CoV-2 and Anti-Glycan Antibodies
Source: Viruses. 2023 Jul 20;15(7):1584. doi: 10.3390/v15071584 (PMC10384250; doi:10.3390/v15071584)
Supplement: Supplementary file 1 [file viruses-15-01584-s001.zip › viruses-2491227-Supplementary SII.pdf]

Supplementary SII

List of ligands

GID	Structure	Common Name
1	Fucα-sp3	-
2	Galα-sp3	-
3	Galβ-sp3	-
4	GalNAcα1-OSer	Tn-Ser
5	GalNAcα-sp3	Tn
6	GalNAcβ-sp3	-
7	Glcα-sp3	-
9	Glcβ-sp3	-
10	GlcNAcβ-sp3	-
11	GlcNAcβ-sp2	-
13	GlcNAcβ-sp8	-
14	GlcN(Gc)b-sp4	-
15	HOCH2(HOCH)4CH2NH2	aminoglucitol
16	Manα-sp3	-
17	Manα-sp4	-
18	Manβ-sp4	-
19	ManNAcβ-sp4	-
20	Rhaα-sp3	-
21	Galβ-sp4	-
22	GlcNAcβ-sp4	-
23	GalNAcβ-sp4	-
24	GlcNAcα-sp3	-
25	GalNAcβ-sp10	-
26	Rhaβ-sp4	-
28	Xylβ-sp4	-
29	Fucβ-sp4	-

30	Glcβ-sp4	-
31	L-Araα-sp4	-
32	GalN(Gc)β-sp3	-
33	Glc-2-NH2	-
34	L-Glcβ-sp4	-
35	GlcNAcβ-sp2	-
37	3-O-Su-Galβ-sp3	-
38	3-O-Su-GalNAcβ-sp3	-
41	6-O-Su-GalNAcα-sp3	-
43	6-O-Su-GlcNAcβ-sp3	-
44	GlcAα-sp3	α-glucuronic acid
45	GlcAβ-sp3	β-glucuronic acid, β-glucuronate
46	6-H2PO3Glcβ-sp4	Glc6P
47	6-H2PO3Manα-sp3	Man6P
48	Neu5Acα-sp3	-
49	Neu5Acα-sp9	-
50	Neu5Acβ-sp3	-
51	Neu5Acβ-sp9	-
52	Neu5Gcα-sp3	-
53	Neu5Gcb-sp3	-
55	3-O-Su-GlcNAcβ-sp3	-
57	D-Ribβ -sp4	-
58	Fucβ-sp3	-
59	αKdo-5-phosphate-sp11	-
60	6-O-Su-Galβ-sp3	-
61	3-O-Su-GalNAcα-sp3	-
62	GlcAβ-sp2	β-glucuronate
63	4-O-Su-GlcNAcβ-sp2	-
64	4-O-Su-Galβ-sp3	-
65	4-O-Su-GalNAcα-sp3	-

66	4-O-Su-GalNAcβ-sp3	-
71	Fucα1-2Galβ-sp3	Hdi
72	Fucα1-3GlcNAcβ-sp3	-
73	Fucα1-4GlcNAcβ-sp3	Le
75	Galα1-2Galβ-sp3	-
76	Galα1-3Galβ-sp3	Bdi
77	Galα1-3GalNAcβ-sp3	-
78	Galα1-3GalNAcα-sp3	Tαα, core 8
80	Galα1-3GlcNAcβ-sp3	-
81	Galα1-4GlcNAcβ-sp3	aLN
82	Galα1-4GlcNAcβ-sp8	aLN
83	Galα1-6Glcβ-sp4	melibiose
84	Galβ1-2Galβ-sp3	-
85	Galβ1-3GlcNAcβ-sp3	Lec
86	Galβ1-3GlcNAcβ-sp2	Lec
87	Galβ1-3Galβ-sp3	-
88	Galβ1-3GalNAcβ-sp3	Tββ
89	Galβ1-3GalNAcα-sp3	TF, core 1
92	Galβ1-4Glcβ-sp2	Lactose, Lac
93	Galβ1-4Glcβ-sp4	Lactose, Lac
94	Galβ1-4Galβ-sp4	-
96	Galβ1-4GlcNAcβ-sp2	N-acetyllactosamine, LN
97	Galβ1-4GlcNAcβ-sp3	N-acetyllactosamine, LN
98	Galβ1-4GlcNAcβ-sp5	N-acetyllactosamine, LN
99	Galβ1-4GlcNAcβ-sp8	N-acetyllactosamine, LN
100	Galβ1-6Galβ-sp4	-
101	GalNAcα1-3GalNAcβ-sp3	Fs-2
102	GalNAcα1-3Galβ-sp3	Adi
103	GalNAcα1-3GalNAcα-sp3	core 5
104	GalNAcβ1-3Galβ-sp3	-

105	GalNAcβ1-3GalNAcβ-sp3	para-Fs
106	GalNAcβ1-4GlcNAcβ-sp3	LacdiNAc
107	GalNAcβ1-4GlcNAcβ-sp2	LacdiNAc
110	Glcα1-4Glcβ-sp3	maltose
111	Glcβ1-4Glcβ-sp4	cellobiose
112	Glcβ1-6Glcβ-sp4	gentiobiose
113	GlcNAcβ1-3GalNAcα-sp3	core 3
114	GlcNAcβ1-3Manβ-sp4	-
116	GlcNAcβ1-4GlcNAcβ-sp3	chitobiose
117	GlcNAcβ1-4GlcNAcβ-sp4	chitobiose
118	GlcNAcβ1-6GalNAcα-sp3	core 6
121	Manα1-4Manβ-sp4	-
122	Manα1-6Manβ-sp4	-
123	Manβ1-4GlcNAcβ-sp4	-
125	6-Bn-Galβ1-4GlcNAcβ-sp2	-
126	6-Bn-Galα1-4(6-Bn)GlcNAcβ-sp3	-
128	Galβ1-4Glcβ-sp4-Trp	-
129	Galβ1-3(6-O-Bn)GlcNAcβ-sp3	-
130	(6-O-Bn-Galβ1)-3GlcNAcβ-sp2	-
131	(6-O-Bn-Galβ1)-3(6-O-Bn)GlcNAcβ-sp3	-
132	Galβ1-3GalNAcα-sp5	TF
133	Galβ1-4Glcβ-sp4-Ala	-
135	Galβ1-4Glcβ-sp4-Asn	-
136	Galβ1-4Glcβ-sp4-Ile	-
137	Galβ1-4Glcβ-sp4-Nle	-
138	Galβ1-4Glcβ-sp4-Val	-
139	Galβ1-4GlcNAcα-sp3	-
140	Galα1-3GalNAc(fur)b-sp3	-
142	GlcNAcα1-3GalNAcβ-sp3	-
143	Fucα1-2(3-O-Su)Galβ-sp3	-

144	Galβ1-3(6-O-Su)GlcNAcβ-sp2	-
145	Galβ1-3(6-O-Su)GlcNAcβ-sp3	-
146	Galβ1-4(6-O-Su)Glcβ-sp2	-
149	GlcNAcβ1-4(6-O-Su)GlcNAcβ-sp2	-
150	3-O-Su-Galβ1-3GalNAcα-sp3	-
151	6-O-Su-Galβ1-3GalNAcα-sp3	-
153	6-O-Su-Galβ1-4Glcβ-sp2	-
154	3-O-Su-Galβ1-3GlcNAcβ-sp3	-
156	3-O-Su-Galβ1-4GlcNAcβ-sp2	-
158	4-O-Su-Galβ1-4GlcNAcβ-sp2	-
159	4-O-Su-Galβ1-4GlcNAcβ-sp3	-
160	6-O-Su-Galβ1-3GlcNAcβ-sp2	-
161	6-O-Su-Galβ1-3GlcNAcβ-sp3	-
162	6-O-Su-Galβ1-4GlcNAcβ-sp2	-
164	GlcAβ1-3GlcNAcβ-sp3	-
165	GlcAβ1-3Galβ-sp3	-
166	GlcAβ1-6Galβ-sp3	-
167	GlcNAcβ1-4-[HOOC(CH3)CH]-3-O-GlcNAcβ-sp4	GlcNAc-Mur
168	GlcNAcβ1-4Mur-L-Ala-D-i-Gln-Lys	GMDP-Lys
169	Neu5Acα2-3Galβ-sp3	GM4
170	Neu5Acα2-6Galβ-sp3	-
171	Neu5Acα2-3GalNAcα-sp3	-
172	Neu5Acα2-6GalNAcα-sp3	SiaTn
173	Neu5Acβ2-6GalNAcα-sp3	-
174	Neu5Gcα2-6GalNAcα-sp3	Neu5GcTn
177	3-O-Su-Galβ1-4(6-O-Su)GlcNAcβ-sp3	-
178	6-O-Su-Galβ1-4(6-O-Su)Glcβ-sp2	-
179	6-O-Su-Galβ1-3(6-O-Su)GlcNAcβ-sp2	-
180	6-O-Su-Galβ1-4(6-O-Su)GlcNAcβ-sp2	-
181	3,4-O-Su2-Galβ1-4GlcNAcβ-sp3	-

182	3,6-O-Su2-Galβ1-4GlcNAcβ-sp2	-
183	4,6-O-Su2-Galβ1-4GlcNAcβ-sp2	-
184	4,6-O-Su2-Galβ1-4GlcNAcβ-sp3	-
186	Neu5Acα2-8Neu5Acα2-sp3	(Neu5Ac)2, (Sia)2
187	Neu5Acα2-8Neu5Acα2-sp9	(Sia)2
188	Neu5Acα2-8Neu5Acβ-sp9	-
189	3,6-O-Su2-Galβ1-4(6-O-Su)GlcNAcβ-sp2	-
190	Galβ1-4-(6-P)GlcNAcβ-sp2	-
191	6-P-Galβ1-4GlcNAcβ-sp2	-
192	GalNAcβ1-4(6-O-Su)GlcNAcβ-sp3	-
193	3-O-Su-GalNAcβ1-4GlcNAcβ-sp3	-
194	6-O-Su-GalNAcβ1-4GlcNAcβ-sp3	-
195	6-O-Su-GalNAcβ1-4(3-O-Ac)GlcNAcβ-sp3	-
196	3-O-Su-GalNAcβ1-4(3-O-Su)GlcNAcβ-sp3	-
197	3,6-O-Su2-GalNAcβ1-4GlcNAcβ-sp3	-
198	4,6-O-Su2-GalNAcβ1-4GlcNAcβ-sp3	-
199	4,6-O-Su2-GalNAcβ1-4-(3-O-Ac)GlcNAcβ-sp3	-
200	4-O-Su-GalNAcβ1-4GlcNAcβ-sp3	-
202	6-O-Su-GalNAcβ1-4(6-O-Su)GlcNAcβ-sp3	-
203	Galβ1-4(6-O-Su)GlcNAcβ-sp2	-
204	4-O-Su-GalNAcβ1-4GlcNAcβ-sp2	-
205	Neu5Acα2-6GalNAcβ-sp3	-
206	Neu5Gcα2-3Galβ-sp3	-
208	Galβ1-3GlcNAcβ-sp4	Lec
209	αKdo-(2→4)-αKdo-sp11	-
211	αKdo-(2→8)-αKdo-sp11	-
213	Neu5Acβ2-6Galβ-sp3	-
214	DGlcAβ1-3Galβ-sp3	ΔGlcAβ3Gal
215	Fucα1-2Galβ1-3GlcNAcβ-sp3	Led, H (type 1)
216	Fucα1-2Galβ1-4GlcNAcβ-sp3	H (type 2)

217	Fucα1-2Galβ1-3GalNAcα-sp3	H (type 3)
219	Fucα1-2Galβ1-4Glcβ-sp4	H (type 6)
221	Galα1-3Galβ1-4Glcβ-sp4	-
222	Galα1-3Galβ1-4GlcNAcβ-sp3	Galili (tri)
223	Galα1-4Galβ1-4Glcβ-sp2	Pk, Gb3, GbOse3
225	Galα1-4Galβ1-4GlcNAcβ-sp2	P1
226	Galα1-3(Fucα1-2)Galβ-sp3	Btri
227	Galα1-3(Fucα1-2)Galβ-sp5	Btri
228	Galβ1-2Galα1-3GlcNAcβ-sp3	-
229	Galβ1-3Galβ1-4GlcNAcβ-sp4	-
230	Fucβ1-2Galβ1-3GlcNAcβ-sp3	-
231	Galβ1-4GlcNAcβ1-3GalNAcα-sp3	-
232	Galβ1-4GlcNAcβ1-6GalNAcα-sp3	-
233	Galβ1-3(Fucα1-4)GlcNAcβ-sp3	Lea
234	Galβ1-4(Fucα1-3)GlcNAcβ-sp3	Lex
235	GalNAcα1-3(Fucα1-2)Galβ-sp3	Atri
236	GalNAcα1-3(Fucα1-2)Galβ-sp5	Atri
237	GalNHα1-3(Fucα1-2)Galβ-OCH2CH2CH2NHAc	ABtri
239	GalNAcβ1-3(Fucα1-2)Galβ-sp3	-
240	(Glcα1-4)3β-sp4	maltotriose
241	(Glcα1-6)3β-sp4	isomaltotriose
242	GlcNAcα1-3Galβ1-4GlcNAcβ-sp2	-
243	GlcNAcα1-3Galβ1-4GlcNAcβ-sp3	-
245	GlcNAcα1-6Galβ1-4GlcNAcβ-sp2	-
246	GlcNAcβ1-2Galβ1-3GalNAcα-sp3	-
247	GlcNAcβ1-3Galβ1-3GalNAcα-sp3	-
249	GlcNAcβ1-3Galβ1-4GlcNAcβ-sp2	-
250	GlcNAcβ1-3Galβ1-4GlcNAcβ-sp3	-
251	GlcNAcβ1-4Galβ1-4GlcNAcβ-sp2	-
252	GlcNAcβ1-4GlcNAcβ1-4GlcNAcβ-sp4	chitotriose

253	GlcNAcβ1-6Galβ1-4GlcNAcβ-sp2	-
254	GlcNAcβ1-6(Galβ1-3)GalNAcα-sp3	core 2
255	GlcNAcβ1-6(GlcNAcβ1-3)GalNAcα-sp3	core 4
256	GlcNAcβ1-6(GlcNAcβ1-4)GalNAcα-sp3	-
258	Manα1-6(Manα1-3)Manβ-sp4	Man3
259	Galβ1-4(Galβ1-3)GlcNAcβ-sp3	-
260	Galβ1-3(Fucβ1-4)GlcNAcβ-sp3	-
263	(GalNAcβ-PEG2)3-β-DD	-
264	Galβ1-4Galβ1-4GlcNAcβ-sp3	-
266	Galα1-4Galβ1-4GlcNAcβ-sp3	P1
267	GlcNAcβ1-3Galβ1-3GlcNAcβ-sp3	-
268	GlcNAcβ1-4(Fucα1-6)GlcNAcβ-sp3	-
272	Neu5Acα2-3Galβ1-4Glcβ-sp4-Cit	-
273	Fucβ1-2Galβ1-4GlcNAcβ-sp3	-
274	GalNAcα1-3Galβ1-4GlcNAcβ-sp3	-
275	GalNAcβ1-3Galβ1-4GlcNAcβ-sp3	-
276	GlcNAcβ1-4Galβ1-4GlcNAcβ-sp3	-
277	GalN(Gc)α1-3(Fucα1-2)Galβ-sp3	Atri(Gc)
278	GalNAcα1-3GalNAcβ1-3Galβ-sp3	Fs-3
279	Galβ1-3GlcNAcα1-3GalNAcα-sp3	LeCa3Tn
280	Galβ1-3GlcNAcβ1-3GalNAcα-sp3	-
281	Fucα1-2Galβ1-3GalNAcβ-sp3	H type4
282	Galβ1-3Galβ1-4GlcNAcβ-sp3	-
283	GlcNAcα1-4Galβ1-4GlcNAcβ-sp3	-
287	3-O-Su-Galβ1-3(Fucα1-4)GlcNAcβ-sp3	3'-O-Su-Lea
288	3-O-Su-Galβ1-4(Fucα1-3)GlcNAcβ-sp3	3'-O-Su-Lex
289	Neu5Acα2-6(Galβ1-3)GalNAcα-sp3	-
290	Neu5Acα2-6(Galα1-3)GalNAcα-sp3	-
291	Neu5Acβ2-6(Galβ1-3)GalNAcα-sp3	-
292	Neu5Acα2-3Galβ1-3GalNAcα-sp3	-

293	Neu5Acα2-3Galβ1-4Glcβ-sp3	3'SL
294	Neu5Acα2-3Galβ1-4Glcβ-sp4	3'SL
295	Neu5Acα2-6Galβ1-4Glcβ-sp2	6'SL
297	Neu5Acβ2-6Galβ1-4Glcβ-sp2	-
298	Neu5Acα2-3Galβ1-4GlcNAcβ-sp3	3'SLN
299	Neu5Acα2-3Galβ1-3GlcNAcβ-sp3	3'SiaLeC
300	Neu5Acα2-6Galβ1-4GlcNAcβ-sp3	6'SLN
302	Neu5Acβ2-6Galβ1-4GlcNAcβ-sp3	-
303	Neu5Gcα2-3Galβ1-4GlcNAcβ-sp3	-
304	Neu5Gcα2-6Galβ1-4GlcNAcβ-sp3	-
305	Neu5Gcβ2-6Galβ1-4GlcNAcβ-sp3	-
309	Neu5Acα2-6(Neu5Acα2-3)GalNAcα-sp3	-
310	Neu5Acα2-3Galβ1-4GlcNAcβ-O(CH2)3NH-amide-Neu5Acα2-3Galβ1-4GlcNAcβ-sp3	-
315	Neu5Acα2-3Galβ1-4-(6-O-Su)GlcNAcβ-sp3	-
316	Fucα1-2(6-O-Su)Galβ1-4GlcNAcβ-sp3	6’-suH(type2)
318	Neu5Acα2-6Galβ1-4(6-O-Su)GlcNAcβ-sp3	-
319	Neu5Acα2-3(6-O-Su)Galβ1-4GlcNAcβ-sp3	-
320	4-O-Su-Neu5Acα2-3(6-O-Su)Galβ1-4GlcNAcβ-sp3	-
321	(Neu5Acα2-8)3-sp3	(Sia)3
322	(Neu5Acα2-8)3b-sp3	-
323	Neu5Acα2-6Galβ1-3GlcNAcβ-sp3	6'-SiaLec
324	Neu5Acα2-6Galβ1-3(6-O-Su)GlcNAcβ-sp3	-
326	Neu5Acα2-3Galβ1-4Glcβ-sp4-Ile	-
327	Neu5Acα2-3Galβ1-4Glcβ-sp4-Nle	-
328	Neu5Acα2-3Galβ1-4Glcβ-sp4-Phe	-
329	Neu5Acα2-3Galβ1-4Glcβ-sp4-Trp	-
331	Neu5Gcα2-3Galβ1-3GlcNAcβ-sp3	-
333	Neu5Gcα2-3Galβ1-3(6-O-Su)GlcNAcβ-sp3	-
334	Neu5Gcα2-3Galβ1-4(6-O-Su)GlcNAcβ-sp3	-
335	Neu5Acα2-3Galβ1-3(6-O-Su)GlcNAcβ-sp3	-

337	GalNAcα1-4Galβ1-4GlcNAcβ-sp3	-
338	Neu5Acα2-6Galβ1-3GalNAcα-sp3	6'-sialyl-TF
339	Neu5Acβ2-6Galβ1-3GalNAcα-sp3	-
340	Galα1-3(Neu5Acβ2-6)GalNAcβ-sp3	-
342	Neu5Acα2-3(6-O-Su)Galβ1-3GalNAcα-sp3	6'Su3'SiaTF
344	Neu5Acβ2-3Galβ1-4GlcNAcβ-sp3	b3’SLN
345	Neu5Acα2-3Galβ1-3(6-O-Su)GalNAcα-sp3	-
346	Neu5Acα2-3-(6-O-Su)Galβ1-4(6-O-Su)GlcNAcβ-sp3	-
347	Galβ1-4(Neu5Acα2-6)GlcNAcβ-sp2	-
348	Neu5Acα2-3Galβ1-4Glcα-sp3	-
349	Neu5Acα2-3(Neu5Acα2-2)Galβ-sp3	-
350	Neu5Gcα2-3Galβ1-4Glcβ-sp3	3'SL(Gc)
359	Galα1-3(Fucα1-2)Galβ1-3GlcNAcβ-sp3	B (type 1)
360	Galα1-3(Fucα1-2)Galβ1-4GlcNAcβ-sp3	B (type 2)
362	Galα1-3(Fucα1-2)Galβ1-3GalNAcα-sp3	B (type 3)
363	Galα1-3(Fucα1-2)Galβ1-3GalNAcβ-sp3	B (type 4)
364	Galα1-3Galβ1-4(Fucα1-3)GlcNAcβ-sp3	αGalLex
365	Galα1-4(Fucα1-2)Galβ1-4GlcNAcβ-sp3	-
366	GalNAcα1-3(Fucα1-2)Galβ1-3GlcNAcβ-sp3	A (type 1)
368	GalNAcα1-3(Fucα1-2)Galβ1-4GlcNAcβ-sp3	A (type 2)
369	GalNAcα1-4(Fucα1-2)Galβ1-4GlcNAcβ-sp3	-
370	GalNAcβ1-3(Fucα1-2)Galβ1-4GlcNAcβ-sp3	-
371	Fucα1-2Galβ1-3(Fucα1-4)GlcNAcβ-sp3	Leb
372	Fucα1-2Galβ1-4(Fucα1-3)GlcNAcβ-sp3	Ley
373	Galα1-3Galβ1-4GlcNAcβ1-3Galβ-sp3	Galili (tetra)
374	Galα1-3(Galα1-4)Galβ1-4GlcNAcβ-sp3	-
375	Galα1-4GlcNAcβ1-3Galβ1-4GlcNAcβ-sp3	-
376	Galβ1-3GlcNAcβ1-3Galβ1-4Glcβ-sp4	N-acetyllacto-N-tetraose, LNT
377	Galβ1-3GlcNAcβ1-3Galβ1-3GlcNAcβ-sp2	-
378	Galβ1-3GlcNAcα1-3Galβ1-4GlcNAcβ-sp3	-

379	Galβ1-3GlcNAcβ1-3Galβ1-4GlcNAcβ-sp3	-
380	Galβ1-3GlcNAcα1-6Galβ1-4GlcNAcβ-sp2	-
381	Galβ1-3GlcNAcβ1-6Galβ1-4GlcNAcβ-sp2	-
383	Galβ1-4GlcNAcβ1-3Galβ1-4Glcβ-sp4	N-acetyllacto-neo-tetraose, LNnT
384	Galβ1-4GlcNAcβ1-3Galβ1-4GlcNAcβ-sp2	i
386	Galβ1-4GlcNAcα1-6Galβ1-4GlcNAcβ-sp2	-
387	Galβ1-4GlcNAcβ1-6Galβ1-4GlcNAcβ-sp2	-
388	Galβ1-4GlcNAcβ1-6(Galβ1-3)GalNAcα-sp3	-
389	GalNAcβ1-3Galα1-4Galβ1-4Glcβ-sp3	Gb4, P
390	(Glcα1-4)4b-sp4	maltotetraose
392	GalNAcα1-3(Fucα1-2)Galβ1-3GalNAcα-sp3	A (type 3)
395	GlcNAcβ1-6(GlcNAcβ1-3)Galβ1-4GlcNAcβ-sp2	Tk
396	(GlcNAcβ1)3-3,4,6-GalNAcα-sp3	-
401	Galβ1-3GlcNAcβ1-3Galβ1-3GlcNAcβ-sp3	-
402	GalNAcα1-3(Fucα1-2)Galα1-4GlcNAcβ-sp3	-
403	Galβ1-3GlcNAcβ1-3Galβ1-4GlcNAcβ-sp2	-
404	GalNAcα1-3Galβ1-4(Fucα1-3)GlcNAcβ-sp3	αGalNAcLex
405	Galα1-3(Fucα1-2)Galα1-4GlcNAcβ-sp3	-
408	GlcNAcβ1-4(GlcNAcβ1-3)Galβ1-4GlcNAcβ-sp3	GlcNAc2-3,4LacNAc
409	GlcNAcβ1-3(GlcNAcα1-6)Galβ1-4GlcNAcβ-sp2	-
410	Galβ1-3GlcNAcα1-3Galβ1-3GlcNAcβ-sp3	-
411	Galβ1-4GlcNAcα1-3Galβ1-4GlcNAcβ-sp3	-
419	3-O-SuGalβ1-4GlcNAcβ1-3Galβ1-4GlcNAcβ-sp3	-
420	4-O-SuGalβ1-4GlcNAcβ1-3Galβ1-4GlcNAcβ-sp3	-
421	Galβ1-3GlcNAcβ1-3Galβ1-4Glcα-sp4	-
423	Neu5Acα2-3Galβ1-4(Fucα1-3)GlcNAcβ-sp3	SiaLex
425	Neu5Acα2-3Galβ1-4(Fucβ1-3)GlcNAcβ-sp3	-
426	Neu5Acα2-3Galβ1-3(Fucα1-4)GlcNAcβ-sp3	SiaLea
428	Neu5Acα2-3Galβ1-4(Fucα1-3)(6-O-Su)GlcNAcβ-sp3	6-O-Su-SiaLex
429	Neu5Acα2-3(6-O-Su)Galβ1-4(Fucα1-3)GlcNAcβ-sp3	6'-O-Su-SiaLex

431	Neu5Acα2-3Galβ1-4(2-O-Su-Fucα1-3)GlcNAcβ-sp3	-
432	Neu5Acα2-3Galβ1-4(3-O-Su-Fucα1-3)GlcNAcβ-sp3	-
434	Neu5Acα2-8Neu5Acα2-3Galβl-4Glcβ-sp4	GD3
435	Neu5Acα2-3Galβ1-4(2-O-Su-Fucα1-3)(6-O-Su)GlcNAcβ-sp3	-
436	4-O-Su-Neu5Acα2-3(6-O-Su)Galβ1-4(Fucα1-3)GlcNAcβ-sp3	-
437	GalNAcα1-3(Fucα1-2)Galβ1-3GalNAcβ-sp3	A(type 4)
438	Fucβ1-2Galβ1-4(Fucα1-3)GlcNAcβ-sp3	-
441	Neu5Acα2-6(Fucα1-2)Galβ1-4GlcNAcβ-sp3	-
442	Neu5Acα2-3(GalNAcβ1-4)Galβ1-4Glcβ-sp4	GM2
479	Fucα1-2Galβ1-3GlcNAcβ1-3Galβ1-4Glcβ-sp4	H(type1), LNFP I
481	Galα1-3Galβ1-4GlcNAcβ1-3Galβ1-4Glcβ-sp4	Galili (penta)
482	Galα1-3(Fucα1-2)Galβ1-3(Fucα1-4)GlcNAcβ-sp3	BLeb
483	Galα1-3(Fucα1-2)Galβ1-4(Fucα1-3)GlcNAcβ-sp3	BLey
485	Galβ1-4GalNAcα1-3(Fucα1-2)Galβ1-4GlcNAcβ-sp3	-
488	Galβ1-4GlcNAcβ1-6(Galβ1-4GlcNAcβ1-3)GalNAcα-sp3	-
489	Galβ1-4GlcNAcβ1-3(GlcNAcβ1-6)Galβ1-4GlcNAcβ-sp2	-
491	GalNAcα1-3(Fucα1-2)Galβ1-4(Fucα1-3)GlcNAcβ-sp3	ALey
492	(Glcα1-6)5b-sp4	isomaltopentaose
493	(GlcNAcβ1-4)5b-sp4	chitopentaose
495	Manα1-6(Manα1-3)Manα1-6(Manα1-3)Manβ-sp4	Man5
496	Fucα1-2Galβ1-3(Fucα1-4)GlcNAcβ1-3Galβ1-4Glcβ-sp4	Leb(hexa)
498	Galβ1-4GlcNAcβ1-3Galβ1-4GlcNAcβ1-3Galβ1-4GlcNAcβ-sp3	(LN)3
499	Galβ1-4GlcNAcβ1-6(Galβ1-4GlcNAcβ1-3)Galβ1-4GlcNAcβ-sp2	I
501	Galβ1-3GalNAcβ1-3Galα1-4Galβ1-4Glcβ-sp4	Gb5
502	(Glcα1-6)6b-sp4	isomaltohexaose
503	(GlcNAcβ1-4)6-sp4	chitohexaose
504	(Aβ1-4GNβ1-2Mα1)2-3,6-Mβ1-4GNβ1-4GNβ-sp4	-
505	(GNβ1-2Mα1)2-3,6-Mβ1-4GNβ1-4GNβ-sp4	-
508	GalNAcβ1-3(Fucα1-2)Galβ1-4(Fucα1-3)GlcNAcβ-sp3	-
511	GalNAcα1-3GalNAcβ1-3Galα1-4Galβ1-4Glcβ-sp4	Fs-5-Gly

512	GalNAcα1-3Galβ1-4GlcNAcα1-3Galβ1-4GlcNAcβ-sp3	-
513	Glcα1-4Glcα1-4Glcα1-4Glcα1-4Glcα1-4Glcβ-sp4	maltohexaose
528	Neu5Acα2-3Galβ1-4(Fucα1-3)GlcNAcβ1-3Galβ-sp3	SiaLex-Gal
534	Neu5Acα2-6Galβ1-4GlcNAcβ1-3Galβ1-4GlcNAcβ-sp3	-
535	Neu5Acα2-8Neu5Acα2-3(GalNAcβ1-4)Galβ1-4Glcβ-sp4	GD2
536	Neu5Aa2-3Galβ1-3GlcNAcβ1-3Galβ1-4Glcβ-sp4	LSTa
537	Neu5Aa2-3Galβ1-4GlcNAcβ1-3Galβ1-4Glcβ-sp4	LSTd
539	Galβ1-4GlcNAcβ1-6(Fucα1-2Galβ1-3GlcNAcβ1-3)Galβ1-4Glcβ-sp4	MFLNH I
627	(Siaα2-6Aβ1-4GNβ1-2Mα1)2-3,6-Mβ1-4GNβ1-4GNβ-sp4	11-OS, YDS
629	Trehalose-ethanolamine	-
630	(GlcAβ1-3GlcNAcβ1-4)$_{20}$-NH(p-C_6H_4)$CH_2CH_2NH_2$	hyaluronic acid
631	(GlcAβ1-3GlcNAcβ1-4)$_{38}$- NH(p-C_6H_4)$CH_2CH_2NH_2$	hyaluronic acid
632	(GlcAβ1-3GlcNAcβ1-4)$_{13}$- NH(p-C_6H_4)$CH_2CH_2NH_2$	hyaluronic acid
633	(Neu5Acα2-8)n- NH(p-C_6H_4)$CH_2CH_2NH_2$	colominic acid
800	GlcNAcα1-4GlcNAcβ-sp3	-
802	Galβ1-3GalNAc(fur)b-sp3	-
804	[Galβ1-4GlcNAcβ-OCH_2CH_2]$_2$NH	-
805	GalNAcβ1-4(6-O-Bn)GlcNAcβ-sp3	-
806	Galα1-6Glcα-sp3	-
808	Galα1-6Glcβ-sp3	melibiose
809	GalNAcβ1-3GalNAcα-sp3	-
810	GalNGcα1-3GalNAcα-sp3	-
811	3,6-O-Me2-Glcβ1-4(2,3-O-Me2)Rhaβ-O(p-C_6H_4)$CH_2CH_2NH_2$	-
812	Galβ1-4Glcα-sp4	-
813	Galβ1-3GalNGcα-sp3	-
814	Manα1-6Manα-sp4	-
815	Galα1-4GalNAcα-sp3	-
816	Galβ1-4GalNAcα-sp3	-
817	GalNAcβ1-4GalNAcα-sp3	-
818	GalNAcα1-4GalNAcα-sp3	-

819	Glcβ1-4GalNAcα-sp3	-
820	GlcNAcβ1-4GalNAcα-sp3	-
821	Galα1-4Galβ-sp3	-
822	GalNAcα1-4Galβ-sp3	-
823	GalNAcβ1-4Galβ-sp3	-
824	Galβ1-4Galβ-sp3	-
825	Glcβ1-3GlcNAcβ-sp3	-
826	Glcβ1-3GalNAcα-sp3	-
827	Glcβ1-3GalNAcβ-sp3	-
828	GlcNAcβ1-2Galβ-sp3	-
829	GlcNAcβ1-4Galβ-sp3	-
830	Glcα1-4GalNAcα-sp3	-
831	Galβ1-4Glcβ-sp3	-
832	Galβ1-4Glcβ-sp4-Leu	-
833	Galβ1-4Glcβ-sp4-Leu-Leu	-
834	Galβ1-4Glcβ-sp4-Leu-Tyr	-
835	Galβ1-4Glcβ-sp4-Leu-Phe	-
836	GlcNAcα1-3Galβ-sp3	-
837	Glcβ1-3Galβ-sp3	-
838	Glcβ1-6Galβ-sp3	-
839	GlcNAcβ1-3Galβ-sp3	-
840	Galβ1-4Glcα-sp3	-
841	Galα1-4Glcβ-sp3	-
842	Galα1-4Glcα-sp3	-
843	Galβ1-3GlcNAcα-sp3	-
850	Galβ1-3(6-O-Su)GalNAcα-sp3	6-suTF
851	Galα1-3(6-O-Su)GalNAcα-sp3	-
852	GlcNAcβ1-4-[HOOC(CH3)CH]-3-O-GlcNAcα-sp4	-
853	6-O-Su-Galα1-3GalNAcα-sp3	-
854	Neu5Acα2-3(6-O-Su)Galβ-sp3	-

855	(O-Su)4Glcα1-4(O-Su)3Glcβ-spacer	-
856	DGlcAβ1-6Galβ-sp3	-
857	Neu5Acα2-8Neu5Acβ-sp3	-
858	Galβ1-4(6-O-Su)Glcβ-sp3	-
859	Estrone-3-GlcA(PEG-NH_2)b	-
900	H-$(Gly)_6$-NH_2 Gly_6-amide, linear	-
901	biot-CMG_2-NH_2	-
908	PEKAXRELC	-
909	VMYAXSGC	-
910	Npentyl-Indol-linked-3-NH_2 (AcOH salt)	-
911	N-C5-indazole-3-CONH-PEG-NH_2 (AcOH salt)	-
912	APPA(Tn)TSGPAPRTDPASTVGHAP-sp12	-
913	APPAT(Lac)YGPAPRTDPASTVGHAP-sp12	-
914	APPATSGPAPRTDPASTVGHAP-sp12	-
915	APPAT(Tn)SGPAPRTDPASTVGHAPPATSG-sp12	-
916	APPAT(SiaTn)SGPAPRTDPASTVGHAP-PEG3-EDAGVPLSVTRVFSGYAFES-PEG3-NH- Ethoxy-cyclobutene-1,2-dione	-
917	APPAT(Tn)SGPAPR(Tn)TDPASTVGHAP-PEG3-NH- Ethoxy-cyclobutene-1,2-dione	-
918	APPAT(Tn)SGPAPR(Tn)TDPASTVGHAP-PEG3-K-PEG3-TALVVDDGVLNEENV-PEG3-NH- Ethoxy-cyclobutene-1,2-dione	-
919	APPAT(Tn)SGPAPR(Tn)TDPASTVGHAP-sp12	-
920	CVKADRPIAISSPGEA	-
921	CVIKEGKDENQGIGYL	-
922	CDKVVSISNSNDDRGL	-
923	CSFDENLQSSPKDRIN	-
925	QRNDGKTRDTNPKC	-
926	CTDAIGPEAEGAKK	-
927	KEEAEKKAEQRALLC	-
928	CGGRSPKSMNEEGASR	-
929	CALQSAKTQKASSDLA	-

931	CMG$_2$ diamine, 2Na-salt	-
932	DLEGKQXNFKNLREFC	CoV2-178
933	KSNLKPFEXDISTEIC	CoV2-458
934	DPSKPSKRXFIEDLLC	CoV2-808
935	CSFKEELXKYFKNHTS	CoV12-1147
936	CKFDEDXSEPVLKGVK	CoV12-1255
937	DLEGKQXNFKNLREFC -PAA(5%)	CoV2-178-PA5
938	KSNLKPFEXDISTEIC -PAA(5%)	CoV2-458-PA5
939	DPSKPSKRXFIEDLLC -PAA(5%)	CoV2-808-PA5
940	CSFKEELXKYFKNHTS -PAA(5%)	CoV12-1147-PA5
941	CKFDEDXSEPVLKGVK --PAA(5%)	CoV12-1255-PA5
942	DLEGKQGNFKNLREFC-PAA(10%)	CoV2-178-PA10
943	KSNLKPFEXDISTEIC-PAA(10%)	CoV2-458-PA10
944	DPSKPSKRXFIEDLLC -PAA(10%)	CoV2-808-PA10
945	CSFKEELXKYFKNHTS -PAA10%)	CoV12-1147-PA10
946	CKFDEDXSEPVLKGVK --PAA(10%)	CoV12-1255-PA10
947	PAA (5%)	К/PA5
948	PAA(10%)	К/PA10
949	PLQSYGXPTNGVGYC	CoV2-495
950	PLQSYGXPTNGVGYHHHHC	CoV2-495H
951	EVRQIAPGQTXKIADC	CoV2-EVR
952	FGAGAALXIPFAMQMC	CoV2-888
953	FGAGAALQIPFAMXMHHHC	CoV2-888H
1001	-4Qui3Nα1-3Rhaα1-4Galβ1-3(Glcβ1-4)GalNα1-	Salmonella enterica O28-deAc
1002	-2Rib-ol5-P-6Galα1-3FucNAmα1-3GlcNβ1-	Salmonella enterica O47-deAc
1003	-4(Fucα1-3)GalNα1-6ManNα1-3Fucα1-3(Glcβ1-4)Galβ1-	Salmonella enterica O16-deAc
1004	-2Fucα1-2Galβ1-3GalNAcα1-3GlcNAcα1-	Salmonella enterica O13
1005	-4Qui3NAcα1-3Rhaα1-4Galβ1-3(Glcβ1-4)GalNAcα1-	Salmonella enterica O28
1006	-4(Fucα1-3)GalNAcα1-6Man2(20%)Ac3(40%)Ac4(20%)Acα1-3Fucα1-3(Glcβ1-4)Galβ1-	Salmonella enterica O16
1007	-2(Galfα1-4)Galα1-3ManNAcβ1-6Galfβ1-3GlcNAcβ1-	Salmonella enterica O17

1008	-3Galf2(30%)Acβ1-3Galα1-	Salmonella enterica O67
1009	-3(S-3HOBut1-2Ala1-4)Qui4Nβ1-6GlcNAcα1-3QuiNAcα1-3GlcNAcα1-	Salmonella enterica O58
1010	-2Manβ1-4Glcα1-3QiuNAcα1-3GlcNAcα1-	Salmonella enterica O41
1011	-3(GalNAcA6NH2α1-2)Rhaα1-2Rhaα1-3Rhaα1-2Rhaα1-3GlcNAcβ1-	Salmonella enterica O62
1012	-2(Fuc3NFoα1-3)Manβ1-3Glcβ1-3GlcNAcβ1-	Salmonella enterica O60
1013	-4Manα1-2Manα1-2Manβ1-3GalNAcα1-	Salmonella enterica O18
1014	-2Galβ1-3GlcNAcα1-4Rhaα1-3GlcNAcβ1-	Salmonella enterica O59
1015	-3(ManNAcβ1-2)Rhaα1-2Rhaα1-2Galα1-3GlcNAcβ1-	Salmonella enterica O42
1016	-2Ribfβ1-4Galβ1-4GlcNAcα1-4Galβ1-3GlcNAcα1-	Salmonella enterica O52
1017	-3GlcNAcβ1-3(Manβ1-4)Galα1-4Rhaα1-	Salmonella enterica O11
1018	-6Glcα1-4(GlcNAcβ1-3)Galβ1-3GalNAcα1-3GlcNAcβ1-	Salmonella enterica O51
1019	-2Glcα1-6Glcα1-4(GlcNAcβ1-3)Galα1-3GlcNAcβ1-	Salmonella enterica O44
1020	-7Neu5Acα2-3FucNAmα1-3GlcNAc6Acβ1-	Salmonella enterica O21
1021	-3(GlcNAcβ1-2)Rhaα1-2Rhaα1-4Glcα1-3GalNAcβ1-	Salmonella enterica O57
1022	-3(Ser2Ac1-4)Qui4Nβ1-3Ribfβ1-4GalNAcα1-3GlcNAcα1-	Salmonella enterica O56
1023	-3(Galβ1-4)Galβ1-4(GlcNAcβ1-2)Glcβ1-3GalNAcβ1-	Salmonella enterica 038
1024	-3(Rha2(%)Ac3(%)Ac4(%)Acα1-4GalAα1-2)Rhaα1-4Glcα1-2Rhaα1-3GlcNAcβ1-	Cronobacter sakazakii G2356 O2
1025	-3FucNAcα1-4(GlcNAcβ1-2)GalAα1-3FucNAcα1-3GlcNAcβ1-	Cronobacter sakazakii G2592 O7
1026	-2(Glcα1-4)Glcβ1-2Fuc3NRHbβ1-6GlcNAcα1-4GalNAcα1-3(Glcα1-6)GlcNAcβ1-	Cronobacter sakazakii G2594 O4
1027	-4Qui3NAcα1-3Rhaα1-6GlcNAcα1-4GlcAβ1-3(Glcβ1-4)GalNAcα1-	Cronobacter sakazakii G2726 O3
1201	-4(Colα1-3)(Colα1-6)Glcα1-4Galα1-3GlcNAcβ1-	Escherichia coli O11
1202	-2Galβ1-3FucNAcα1-3GlcNAcβ1-	Escherichia coli O15
1203	-2Manα1-2(Glcα1-4)Manβ1-3GlcNAcα1-6Manα1-	Escherichia coli O44
1204	-2(S-3HOBut1-4)Qui4Nα1-4GalNAcβ1-4Rhaα1-3GlcNAc6(30%)Acβ1-	Escherichia coli O49
1205	-3(GlcNAcβ1-2)Rhaα1-2Rhaα1-4Glcα1-3GalNAcβ1-	Escherichia coli O51
1206	-3Fucf2(50%)Acβ1-3-6dmanHepβ1-	Escherichia coli O52
1207	-2-DRha4NAcα1-3Fucα1-4Glcβ1-3GalNAcα1-	Escherichia coli O57
1208	-4(R-Lac2-3Rhap2Acα1-3)Manβ1-4Manα1-3GalNAcβ1-	Escherichia coli O58
1209	-4Quip3NAcα1-3Rhaα1-4Galβ1-3GalNAcα1-	Escherichia coli O71
1210	-6Manα1-2(Glcα1-4)Manα1-2(Glcα1-3)Manβ1-3GlcNAcα1-	Escherichia coli O73

1211	-2(Galfα1-4)Galα1-3ManNAcβ1-6Galfb1-3GlcNAcβ1-	Escherichia coli O85
1212	-3)Fucα1-3Xlufb1-	Escherichia coli O95
1213	-3(Glcα1-2)Rhaα1-2Rhaα1-2Rhaα1-3(Glcα1-2)Rhaα1-	Escherichia coli O99
1214	-4-8eLeg5Ac7Acα2-6Galα1-3FucNAcα1-3GlcNAcα1-	Escherichia coli O108
1215	-4(GlcpNAcβ1-3)GalNAcα1-2Glcα1-4L-IdoAα1-3GalNAcβ1-	Escherichia coli O112ab
1216	-3Rib-ol5-P-6Galα1-3FucNAma1-3GlcNAcβ1-	Escherichia coli O118
1217	-2(RhaNAc3NFob1-3)Manβ1-3Galα1-4Rhaα1-3GlcNAcα1-	Escherichia coli O119
1218	-3(S-3HOBut1-2DAla1-4)Qui4Nb1-6GlcNAcα1-3LQuiNAcα1-3GlcNAc6(30%)Acα1-	Escherichia coli O123
1219	-2(Glcα1-3)Manα1-3Fucα1-3GalNAcα1-4(Galβ1-3)GalNAcβ1-	Escherichia coli O125
1220	-2Fuc3(65%)Ac4(35%)Acα1-2Galβ1-3GalNAcα1-3GalNAcα1-	Escherichia coli O127
1221	-4(GalNAcβ1-3)Galα1-6Glcβ1-3GalNAcβ1-	Escherichia coli O130
1222	-3Rhaα1-3Rhaα1-2Glcα1-3GlcNAcα1-	Escherichia coli O148
1223	-3(S-Lac2-4)GlcNAcβ1-2Rhaα1-2Rhaα1-3(Glcβ1-2)Rhaα1-3GlcNAcβ1-	Escherichia coli O150
1224	-2Rib-ol5-P-6Galα1-3FucNAma1-3(GlcNAcβ1-4)GlcNAcβ1-	Escherichia coli O151
1225	-8(D-Ala1-7)Leg5Acα2-4GlcAβ1-3GlcNAcβ1-	Escherichia coli O161
1226	-4(Fucα1-3)GlcNAc6(30%)Acα1-4GlcAα1-3Fucα1-3GlcNAcβ1-	Escherichia coli O168
1227	-2Galβ1-4Manβ1-4Galα1-3GlcNAcβ1-	Escherichia coli O40
1228	-2)Galα1-3(Fucα1-2)Galβ1-3GalNAcβ1-3GalNAcβ(1-	Escherichia coli O86:B7
1230	Escherichia coli O10a10b	Escherichia coli O10a10b
1231	-2Glcβ1-6GlcNAcα1-3FucNAcα1-3GlcNAcβ1-	Escherichia coli O12
1232	Galα1-2Galα1-2(Galβ1-4)Glcα1-3Glcα1-/inner core-lipid A/	Escherichia coli O14
1233	-2Rhaα1-2Rhaα1-2Rhaα1-2Glcα1-3GlcNAc6Acα1-	Escherichia coli O19ab
1234	Escherichia coli O27	Escherichia coli O27
1236	-4(D-Gro1-P-O-3)GalNAcβ1-3Galα1-4Galβ1-3GalNAcβ1-	Escherichia coli O37
1237	-3(R-3HOBut1-4)Qui4Nb1-4(Galα1-3)Manα1-4Rhaα1-3GlcNAcα-	Escherichia coli O39
1238	-3Galα1-3(GlcAβ1-4)Fucα1-4GlcNAcβ1-3Fucα1-3GlcNAcβ1-	Escherichia coli O41
1239	Escherichia coli O54	Escherichia coli O54
1240	Escherichia coli O62	Escherichia coli O62
1241	Escherichia coli O81	Escherichia coli O81
1242	-4GlcAβ1-4(GlcNAcβ1-2)GlcAβ1-3GlcNAcα1-	Escherichia coli O30

1243	-4(Rhaα1-2Fucα1-3)Manα1-3Fucα1-3GlcNAcβ1-	Escherichia coli O36
1246	-4(D-aThr3(70%)Ac2-6)GlcAβ1-6Galβ1-6Glcβ1-3GalNAc6(15%)Acb1-	Escherichia coli O46
1247	-6(Rhaα1-3)Manα1-2(Glcα1-3)Manα1-2Manα1-2Manβ1-3GlcNAcα1-	Escherichia coli O68
1248	GlcNAcα1-2Glcα1-2Glcα1-3(Galα1-6)Glcα1-/inner core-lipid A/	Escherichia coli O100
1249	Escherichia coli O102	Escherichia coli O102
1250	-3(Rhaα1-4)GlcAα1-2Rhaα1-2Rhaα1-2Galα1-3GalNAcβ1-	Escherichia coli O120
1251	-2Rhaα1-2Rhaα1-3Rha2Acα1-3(Glcα1-6)GlcNAcβ1-	Escherichia coli O135
1252	-3(Galfα1-2Rhaα1-4)Galβ1-4Glcα1-4GlcAα1-3GalNAcβ1-	Escherichia coli O140
1253	-2Ribfβ1-4Galβ1-4GlcNAcα1-4Galβ1-3GlcNAcα1-	Escherichia coli O153
1254	-2(ManNAcα1-3)Rhaα1-3Rhaα1-3Rhaα1-3GalNAcα1-	Escherichia coli O154
1255	-2Rha4NAcα1-3Fucα1-4Glcβ1-3GalNAcα1-	Escherichia coli O157
1256	-4(Rhaα1-3)(Glcα1-6)Glcα1-3GalNAcα1-3GalNAcβ1-	Escherichia coli O158
1257	-2Manβ1-4GlcAβ1-3LQuiNAcα1-3GlcNAcα1-	Escherichia coli O163
1258	-4ManNAc3NAcAβ1-2Rhaα1-3Rhaβ1-4GlcNAcα1-	Escherichia coli O180
1259	Escherichia coli O84-deAc	Escherichia coli O84-deAc
1301	-3(Qui3NFoα1-4)GalA6NH2α1-4GalNAcα1-4Galα1-3GalNAcβ1-	Providencia alcalifaciens O3
1302	Providencia alcalifaciens O3_2 capsular polysaccharide	Providencia alcalifaciens O3_2 capsular polysaccharide
1303	-4GlcNAcβ1-3GlcAα1-4GlcNAcα1-3Rha2Acb1-	Providencia alcalifaciens O7
1304	-2Glcβ1-6Galα1-6GalNAcα1-4(Glcβ1-3)GalNAcα1-3GalNAcα1-	Providencia alcalifaciens O9
1305	Providencia rustigianii O11 capsular polysaccharide	Providencia rustigianii O11 capsular polysaccharide
1306	-4(GlcNAcβ1-2Glcβ1-2)(GlcNAcβ1-3)Galβ1-3GalNAcα1-4Galβ1-3GalNAcβ1-	Providencia alcalifaciens O12
1307	-4(D-GroA1NH2(2-P-3))GalNAcβ1-4Galβ1-3FucNAc4Nb1-	Providencia alcalifaciens O22
1308	-3(Dhpa2-4Manβ1-4)Galα1-4GalNAcβ1-3GalNAcβ1-	Providencia alcalifaciens O31
1309	-4Qui3NFob1-3Galα1-3GlcAβ1-3GalNAcβ1-	Providencia alcalifaciens O40
1310	-3GlcAβ1-4(Glcα1-3)Fucα1-4Fucα1-2Glcβ1-3GlcNAcα1-	Providencia alcalifaciens O46-deAc
1311	-3Manα1-2Fucα1-2GlcA4Acb1-3GalNAcα1-	Providencia alcalifaciens O48
1312	-4Glcβ1-3Galα1-4GalNAcβ1-4(L-Ser2-6)GlcAβ1-3GalNAcβ1-	Providencia alcalifaciens O60
1401	GalNAcα1-4GlcNAc3NAcAβ1-3DFucNAcα1-3QuiNAcβ1-3Rhaα1-3(Glcα1-6)Glcβ1-3(Glcα1-	Pseudomonas aeruginosa O1(F4)

	4)(L-Ala1-2)GalNα(P-6)1-3L-gro-manHepp7Cma1-3(P-2)(P-4)L-gro-manHepa1-5Kdoa	
1402	-3Rhaα1-4LGalNAcAα1-3QuiNAcα1-	Pseudomonas aeruginosa O10ac(F5)
1403	-2LGlcβ1-3FucNAcα1-3DFucNAcβ1-	Pseudomonas aeruginosa O11ab
1404	-2Rhaα1-3Rhaα1-4GalNAcA3Acα1-3QuiNAcβ1-	Pseudomonas aeruginosa O13ab
1405	Rhaα1-3Rhaα1-4GalNAcAα1-3QuiNAcβ1-3Rhaα1-3(Glcα1-6)Glcβ1-3(Glcα1-4)(L-Ala1-2GalNα1-3(P-6)L-gro-manHep7Cma1-3(P-2)(P-4)L-gro-manHepa1-5Kdoa	Pseudomonas aeruginosa O13ac
1406	Glcα1-6Glcβ1-3(Rhaα1-6Glcα1-4)GalNα1-3(P-6)L-gro-manHepa1-3(P-2)(P-4)L-gro-manHepa1-5(Kdoa2-4)Kdoa2-6(P-4)GlcNb1-6GlcNa1-P	Pseudomonas aeruginosa O14
1407	-2Ribfβ1-3GalNAcα1-	Pseudomonas aeruginosa O15
1408	Pseudomonas aeruginosa O2abc	Pseudomonas aeruginosa O2abc
1409	-4ManNAc3NAmAβ1-4LGulNAc3NAcAα1-3DFucNAc4Nb1-	Pseudomonas aeruginosa O2ac
1410	-4ManNAc3NAmAβ1-4LGulNAc3NAcAα1-3DFucNAcβ1-	Pseudomonas aeruginosa O2ac(F3)
1411	-4ManNAc3NAmAβ1-4ManNAc3NAcAβ1-3DFucNAcα1-	Pseudomonas aeruginosa O2ad(F7)
1412	-4LGulNAc3NAmAα1-4ManNAc3NAcAβ1-3DFucNAc4Acα1-	Pseudomonas aeruginosa O2adf
1413	-2LRha3Acα1-6GlcNAcα1-4LGalNAcA3Acα1-3(S-3HOBut1-4)QuiNAc4Nb1-	Pseudomonas aeruginosa O3(Habs 3)
1414	-2Rha3Acα1-6GlcNAcα1-4LGalNAcAα1-3QuiNAc4NSHbβ1-	Pseudomonas aeruginosa O3ab
1415	-2Rhaα1-3FucNAcα1-3FucNAcα1-3QuiNAcα1-	Pseudomonas aeruginosa O4ab
1416	-2Rhaα1-3FucNAcα1-3FucNAcα1-3DFucNAcα1-	Pseudomonas aeruginosa O4ac
1417	-2Rhaα1-4GalNAcA3Acα1-4GalNFoAα1-3QuiNAcα1-	Pseudomonas aeruginosa O6(F1)
1418	-3R-3HOBut1-7Pse4Ac5Acβ2-4DFucNAcα1-3QuiNAcβ1-	Pseudomonas aeruginosa O9ad
1419	Pseudomonas aeruginosa O10a10b	Pseudomonas aeruginosa O10a10b
1501	Proteus genomospecies 5/6 O79	Proteus genomospecies 5/6 O79
1502	-3GalNAcβ1-4(L6dTala1-3)Manα1-3L6dTala1-	Aeromonas hydrophila O34-deAc
1503	-2Rhaα1-2Rhaα1-2Rhaα1-4GalAα1-3GlcNAcα1-	Enterobacter cloacae G2277
1504	-3Rhaβ1-4(Glcα1-3)Rhaα1-2Rhaα1-3Galα1-3DFucNAcα1-	Enterobacter cloacae G3421
1601	Proteus mirabilis 12B-r	Proteus mirabilis 12B-r
1602	-3(Rib1(50%)Ac-ol5-P-6)Galβ1-4(GlcNAcβ1-2)Glcβ1-3GlcNAcβ1-	Proteus mirabilis 1B-m
1603	-2Glcβ1-3L6dTal2(85%)Acα1-3GlcNAcβ1-	Proteus mirabilis 3B-m
1604	-4(LAltpAα1-3)GalNAcα1-3GalAα1-3GlcNAcα1-	Proteus mirabilis HJ 4320
1605	-3(Glcα1-6)GlcNAcβ1-4(GlcNAcβ1-2)GlcAβ1-3(L-Thr2-6)GalAβ1-	Proteus mirabilis O11

1606	-3GlcNAcβ1-3(S,R-CetLys2-6GalAα1-4)Galα1-	Proteus mirabilis O13
1607	GalNAcβ1-4GalNAcα1-3GlcNAcα1-2Rib-ol	Proteus mirabilis O16
1608	-3GlcAβ1-4(Galα1-3)FucNAcα1-3GlcNAcα1-	Proteus mirabilis O23
1609	-4(Lys2-6)GalAα1-4Galα1-3(Ser-(2-6)GalA4Acα1-3GlcNAcβ1-	Proteus mirabilis O28
1610	-3LQuiNAcα1-3GlcNAcα1-6(S-Lac-1-3)GlcNAcα1-	Proteus mirabilis O31
1611	-2(Rib-ol5-P-3)Galβ1-3GlcNAcα1-3(EtN1-75%P-6)Glcβ1-3GlcNAcβ1-	Proteus mirabilis O33
1612	-2Fuc3N(R-3HOBu)4Acb1-6Glc3Acα1-4GlcAβ1-3GlcNAcα1-	Proteus mirabilis O35
1613	-3(EtNAc1-P-6)GlcNAcα1-3D-Asp2Ac4-4)Qui4Nb1-6Glcα1-4GalAα1-	Proteus mirabilis O38
1614	-6(GalA6(L-Lys)a1-4)GalNAcβ1-4(Glcα1-2)GlcAβ1-3GalNAcβ1-	Proteus mirabilis O3ab
1615	-4(GalA6(L-Thr)3Acα1-3)GalNAcβ1-3Rhaβ1-4GlcNAc6Acb1-	Proteus mirabilis O58
1616	-4(GlcAα1-3)FucNAcα1-3GlcNAcβ1-	Proteus mirabilis O6
1617	-4(S,R-CetLys2-6)GlcAβ1-6GalNAcα1-6GlcNAcβ1-3GlcNAcβ1-	Proteus mirabilis O60
1618	-6GlcNAcα1-3Galβ1-3GalNAcα1-	Proteus mirabilis OE
1701	-3GlcAβ1-4(Galα1-3)FucNAcα1-3GlcNAcα1-	Proteus penneri 107
1702	-3(Glcβ1-3GlcNAc4(S-Lac)b1-2)Rhaα1-2Rhaα1-2Gal6Acα1-3GlcNAcβ1-	Proteus penneri 113
1703	-4(Glcα1-2)GlcA3Acb1-3GlcNAcα1-2(R-3HOBut1-3)Fuc3Nb1-6Glc4Acα1-	Proteus penneri 17
1704	-6GlcNAc3(S-Lac)a1-3LQuiNAcα1-3GlcNAcα1-	Proteus penneri 28
1705	-3Galα1-4GalNAcα1-3FucNAcα1-3(EtN1-P-6)GlcAcb1-	Proteus penneri 31
1706	-6(S-Lac2-3)GlcNAcβ1-3Galα1-3GlcNAc6Acb1-	Proteus penneri 40
1707	-4(Glcα1-3)Glcβ1-3Galβ1-3GalNAcβ1-4Rib-ol5-P-	Proteus penneri 75
1801	-3(EtN1-P-6)GlcNAcα1-2(R-3HOBut1-3)Fuc3Nb1-6Glcα1-4GlcAβ1-	Proteus vulgaris 32/57 O17
1802	-4(L-Ala2-6)GlcAβ1-3GalNAcβ1-4Glcβ1-3Galα1-4GalNAcβ1-	Proteus vulgaris 70/57 O44
1803	-4GalN6Acα1-3DFuc2Acα1-3(EtN1-P-6)GlcNAcβ1-3Galα1-	Proteus vulgaris O19ab
1804	-3GlcNAcβ1-3(Qui3NAc2(65%)Ac4Acα1-2)Rhaβ1-4Rhaα1-4GlcAβ1-	Proteus vulgaris O22
1805	-4GalNAcβ1-3GlcNAcβ1-2(R-Lac1-3Glcα1-3)Rhaα1-2Ribfβ1-	Proteus vulgaris O25
1806	-4GlcAβ1-3GlcNAcβ1-2(R-3HOBut1-2L-Ala1-4)Qui4Nb1-3Galα1-	Proteus vulgaris O4
1807	-4Glc6(65%)Acα1-3GlcA4Acb1-3GlcNAcα1-3GlcA4(87%)Acb1-	Proteus vulgaris O46
1808	-4Glcβ1-3GalNAcβ1-4GalNAcβ1-4Galβ1-	Proteus vulgaris O65
1809	-4LQuiNAcα1-3GlcNAcα1-4(LQuiNAcα1-3)GalNAcα1-4Galα1-P	Proteus vulgaris OX19
1810	-4Glcβ1-3GalNAcβ1-4GalNAcβ1-4Galβ1-	Proteus vulgaris TG251

1901	Acinetobacter baumannii LUH5534	Acinetobacter baumannii LUH5534
2001	-3(Ribfβ1-4GlcAβ1-4)Galα1-6Manα1-2Manα1-3GalNAcβ1-	Shigella boydii type 10
2002	-3GlcNAcβ1-4GlcA3Acb1-2(Rha3Acα1-3)Manα1-4Galβ1-	Shigella boydii type 12
2003	-6Galα1-4GlcAβ1-6Galβ1-4Galβ1-4GlcNAcβ1-	Shigella boydii type 14
2004	-4(GlcNAcβ1-3)GalNAcα1-4Glcα1-4L-IdoAα1-3GalNAcβ1-	Shigella boydii type 15
2005	-4GlcAβ1-2(Galα1-3)Man6(50%)Acb1-4Manβ1-3GlcNAcβ1-	Shigella boydii type 16
2006	-6(R-Lac2-4)Glcβ1-4GalNAcα1-3GalNAcβ1-	Shigella boydii type 17
2007	-3Rhaβ1-4Rhaα1-2Rhaα1-2GalAα1-3GalNAcα1-	Shigella boydii type 18
2008	-2Ribfβ1-4GalAα1-3GlcNAcα1-2(Galfb1-3)Rhaα1-2Rhaα1-2Ribfβ1-4GalAα1-	Shigella boydii type 2
2009	-3(GlcAβ1-4)Galα1-6Manα1-2Manα1-3GalNAcβ1-	Shigella boydii type 6
2010	-2Galfβ1-3GlcNAcα1-8(3HOBut1-7)Pse5Ac2-6Galα1-6Glcα1-	Shigella boydii type 7
2011	-3GalNAcα1-4GlcAβ1-3GlcNAcβ1-2GalAβ1-	Shigella boydii type 8
2012	-4Glcα1-4GlcAβ1-3GlcNAcα1-3Rhaα1-	Shigella boydii type 9
2013	Shigella boydii type X	Shigella boydii type X
2101	-3Rhaα1-3Rhaα1-2Galα1-3GlcNAcα1-	Shigella dysenteriae type 1
2102	-1D-Gro3-P-6Glcβ1-4(Glcα1-6Gal2(25%)Acα1-3)FucNAcα1-3GlcNAcβ1-	Shigella dysenteriae type 11
2103	-3(R-Lac2-4Glcβ1-6Glcα1-4)Galβ1-6Galfb1-3GalNAcβ1-	Shigella dysenteriae type 3
2104	-3GlcNAcα1-3(Fuc3Ac4Acα1-4)GlcNAcα1-4GlcAα1-3Fucα1-	Shigella dysenteriae type 4
2105	GalNAcA3Ac6NH2α1-4GalNAcAα1-3GlcNAc	Shigella dysenteriae type 7
2106	-4GlcAβ1-3GalNAcβ1-3(GlcNAcβ1-4Glcβ1-4)GalNAcβ1-	Shigella dysenteriae type 8
2107	-2Gal3,4(RPyr)b1-4Manβ1-4Galα1-3GlcNAcβ1-	Shigella dysenteriae type 9
2201	-3GlcNAcβ1-2Rhaα1-2Rhaα1-3(Glcα1-4)Rhaα1-	Shigella flexneri type 2a
2202	-3GlcNAcβ1-2(Glcα1-3)Rhaα1-2Rhaα1-3(Glcα1-4)Rhaα1-	Shigella flexneri type 2b
2203	-3GlcNAcβ1-2(Glcα1-3)Rhaα1-2Rhaα1-3Rha2Acα1-	Shigella flexneri type 3a
2204	-2Rhaα1-2Rhaα1-3Rha2Acα1-3GlcNAcβ1-	Shigella flexneri type 3b
2205	-3(Glcα1-6)GlcNAcβ1-2Rhaα1-2Rhaα1-3Rhaα1-	Shigella flexneri type 4a
2206	-3(Glcα1-6)GlcNAcβ1-2Rhaα1-2Rhaα1-3Rha2Acα1-	Shigella flexneri type 4b
2207	-3GlcNAcβ1-2Rhaα1-2(Glcα1-3)Rhaα1-3Rhaα1-	Shigella flexneri type 5a
2208	-3GlcNAcβ1-2(Glcα1-3)Rhaα1-2(Glcα1-3)Rhaα1-3Rhaα1-	Shigella flexneri type 5b
2209	-2Rha3(%)Ac4(%)Acα1-2Rhaα1-4GalAβ1-3GalNAcβ1-	Shigella flexneri type 6

2210	-2Rha3(60%)Ac4(30%)Acα1-2Rhaα1-4GalAβ1-3GalNAcβ1-	Shigella flexneri type 6b
2211	-3GlcNAcβ1-2(Glcα1-3)Rhaα1-2Rhaα1-3Rhaα1-	Shigella flexneri type X
2212	-2Rhaα1-2Rhaα1-3Rhaα1-3GlcNAcβ1-	Shigella flexneri type Y
2213	-3Rhaα1-3(Glcα1-4)GlcNAcβ1-2Rhaα1-2Rhaα1-	Shigella flexneri type 1a
2214	Shigella flexneri type 2c	Shigella flexneri type 2c
2215	-2(EtN1-P-3)Rhaα1-2Rhaα1-3Rhaα1-3(Glcα1-6)GlcNAcβ1-	Shigella flexneri 4av
2216	Shigella flexneri type 5c	Shigella flexneri type 5c
2217	-2(Glcα1-3)Rhaα1-2Rhaα1-3Rhaα1-3(Glcα1-4)GlcNAcβ1-	Shigella flexneri type 1d
2219	-2Rha3(%)Acα1-2Rhaα1-3Rhaα1-3GlcNAc6(%)Acb1-	Shigella flexneri Y_2
2220	-2(EtN1-P-3)Rhaα1-2(20%EtN1-P-3)Rhaα1-3Rhaα1-3GlcNAc6(45%)Acb1-	Shigella flexneri Yv
2221	-2(Glcα1-3)Rhaα1-2(EtN1-P-3)Rhaα1-3Rhaα1-3GlcNAcβ1-	Shigella flexneri Xv
2501	Streptococcus equi sp. hyaluronic acid sodium salt	Streptococcus equi sp. hyaluronic acid sodium salt
2502	-4(2-O-Su)IdoAα1-4(6-O-Su)GlcNSua1-; -4GlcAβ1-4GlcNAcα1-	Heparin, MW7400
2601	Acetobacter methanolicus LPS	Acetobacter methanolicus LPS
3001	-3Glcβ1-	Saccharomyces cerevisiae zymozan A
3002	-6Manα1-	Yeast mannan (a)
3301	-3Glcβ1-6Glcβ1-	Laminaria hyperborea laminarin
3302	-3Glcβ1-	B5-2 Laminaran
3401	-4Glcβ1-4Glcβ1-3Glcβ1-	Barley β-Glucan
3501	Rha:Ara:Gal:GalA 9:3:79:9, MW 900-2000 kDa	Galαctan-1 from flax bast fibre
3502	Rha:Ara:Gal:GalA 17:3:62:18, MW 100-400kDa	Galαctan-2 from flax bast fibre
8001	-2)Galα1-3(Fucα1-2)Galβ1-3GalNAcβ1-3GalNAcβ(1-LPS	Escherichia coli O86:B7 LPS
8002	-3Rhaα1-3Rhaα1-2Glcα1-3GlcNAcα1- LPS	Escherichia coli O148 LPS
8003	-4(Fucα1-3)GlcNAc6(30%)Acα1-4GlcAα1-3Fucα1-3GlcNAcβ1- LPS	Escherichia coli O168 LPS
8004	-4(GalNAcβ1-3)Galα1-6Glcβ1-3GalNAcβ1- LPS	Escherichia coli O130 LPS
8005	-2Rib-ol5-P-6Galα1-3FucNAma1-3(GlcNAcβ1-4)GlcNAcβ1- LPS	Escherichia coli O151 LPS
8006	-3(Glcα1-2)Rhaα1-2Rhaα1-2Rhaα1-3(Glcα1-2)Rhaα1- LPS	Escherichia coli O99 LPS
8007	-6Manα1-2(Glcα1-4)Manα1-2(Glcα1-3)Manβ1-3GlcNAcα1- LPS	Escherichia coli O73 LPS
8008	-3Rib-ol5-P-6Galα1-3FucNAma1-3GlcNAcβ1- LPS	Escherichia coli O118 LPS

8009	-4(R-Lac2-3Rhap2Acα1-3)Manβ1-4Manα1-3GalNAcβ1- LPS	Escherichia coli O58 LPS
8010	-4(GlcpNAcβ1-3)GalNAcα1-2Glcα1-4L-IdoAα1-3GalNAcβ1- LPS	Escherichia coli O112ab LPS
8011	-3(S-Lac2-4)GlcNAcβ1-2Rhaα1-2Rhaα1-3(Glcβ1-2)Rhaα1-3GlcNAcβ1- LPS	Escherichia coli O150 LPS
8012	Escherichia coli O40 LPS	Escherichia coli O40 LPS
8013	Escherichia coli O49 LPS	Escherichia coli O49 LPS
8014	Escherichia coli O81 LPS	Escherichia coli O81 LPS
8015	Escherichia coli O52 LPS	Escherichia coli O52 LPS
9001	-3Glcβ1-3Glcβ1-3(Glcβ1-6)Glcβ1-	Scleroglucan
9002	-3Glcβ1-	Curdlan
9003	-4GlcN(%)Acb1-	Chitosan, high MW
9004	Saccharomyces cerevisiae mannan SIGMA	-
9005	Bakers yeast glucan SIGMA	-
9006	Saccharomyces cerevisiae zymosan A SIGMA	-
9007	Hyaluronic acid E77-2 KBL6413 17kDa	-
9008	-4GlcAβ1-3(6-O-Su)GalNAcβ1-	Hondroitin-6-Sulfate Sodium salt, MW 60000
10013	N-protein SARS-CoV-2, Xema, 1 ug/ml	RE053N-1
10014	N-protein SARS-CoV-2, Xema, 10 ug/ml	RE053N-10
10015	RBD S-protein SARS-CoV-2, Xema, 1 ug/ml	RH053R-1
10016	RBD S-protein SARS-CoV-2, Xema, 10 ug/ml	RH053R-10

Abbreviation:

sp1 = ol = aminoalditol

sp2 = C2 = $-O(CH_2)_2NH_2$

sp3 = C3 = $-O(CH_2)_3NH_2$

sp4 =Gly = $-NHCOCH_2NH_2$

sp5 =C8 = $-O(CH_2)_3NH-CO(CH_2)_5NH_2$

sp6 = $NH(p\text{-}C_6H_4)CH_2CH_2NH_2$ (as-aminoalditol)

sp7 = Ph = $-OC_6H_4\text{-p-}NH_2$

sp8 = PEG = $-(OCH_2CH_2)_6NH_2$

sp9 = Bn = -$OCH_2C_6H_4$-p-NHCOCH2NH2
sp10 = PEG2 = -$(OCH_2CH_2)_2NH_2$
sp11 = -$OCH_2CH_2CH_2SCH_2CH_2NH_2$
sp12 = PEG3 = H_2N-$(CH_2CH_2O)_3$-CH_2CH_2CO
LPS - lipopolysaccharide
CPS - capsular polysaccharide
sp0 = other spacers:Asn, Ser, C2Et (see details in the column "Spacered form of saccharide")
Ala = alanine
Arg = arginine
Asn = asparagine, NH-CO-$CH_2CH(COOH)NH_2$
Ile = iso-leucine
Nle = norleucine
Ser = serine, -$OCH_2CH(COOH)NH_2$
Trp = tryptophan
Phe = phenylalanine, -$COCH(CH_2C_6H_5)NH_2$
C2Et = -$O(CH_2)_2$NHEt
Cit = citrullin
A =
Gal
AN = GalNAc
Ch = chito
F = L-Fuc
G =
Glc
Gc = glycolyl
GN = GlcNAcβ
R =
Rha
i = iso
(fur, f) = furanose cycle
Fm = formyl

Lac = lactose
LN = N-acetyllactosamine
M = Man
MN = ManNAc
Malt = maltose
OS = oligosaccharide
LPS = lipopolysaccharide
P = phosphate
S = Sia = Neu5Acα
Su = sulfate
Tn = GalNAcα
U = uronic acid
D-Fuc3N*R*Hb = 3,6-dideoxy-3-[(*R*)-3-hydroxybutanoyl]-D-galactose
D-Xlu = D-*threo*-pent-2-ulose (D-xylulose)
8eLeg = 8-epilegionaminic acid
8-Leg = 8-legionaminic acid
L-Ido = L-iduronic acid
D-aThr = D-allothreonine
Dhpa = (2R,4R)-2,4-dihydroxypentanoic acid
L-6dTal = 6-deoxy-L-talose
D-QuiNAc – N-acetyl-D-quinovosamine
Pse - pseudaminic acid
KDO - 3-deoxy- d-manno-oct-2-ulosonic acid or keto-deoxyoctulosonate
6dmanHep - 6-deoxy-D-manno-heptose
Aci - acinetaminic acid
